# ALK upregulates POSTN and WNT signaling to drive neuroblastoma

**DOI:** 10.1016/j.celrep.2024.113927

**Published:** 2024-03-06

**Authors:** Miller Huang, Wanqi Fang, Alvin Farrel, Linwei Li, Antonios Chronopoulos, Nicole Nasholm, Bo Cheng, Tina Zheng, Hiroyuki Yoda, Megumi J. Barata, Tania Porras, Matthew L. Miller, Qiqi Zhen, Lisa Ghiglieri, Lauren McHenry, Linyu Wang, Shahab Asgharzadeh, JinSeok Park, W. Clay Gustafson, Katherine K. Matthay, John M. Maris, William A. Weiss

**Affiliations:** 1 Children’s Hospital Los Angeles, Cancer and Blood Disease Institutes, and The Saban Research Institute, Los Angeles, CA, USA; 2 Keck School of Medicine, University of Southern California, Los Angeles, CA, USA; 3 Division of Oncology and Center for Childhood Cancer Research, Children’s Hospital of Philadelphia, Philadelphia, PA, USA; 4 Department of Biomedical and Health Informatics, Children’s Hospital of Philadelphia, Philadelphia, PA, USA; 5 Department of Neurology, University of California, San Francisco, San Francisco, CA, USA; 6 Helen Diller Family Comprehensive Cancer Center, University of California, San Francisco, San Francisco, CA, USA; 7 Departments of Pediatrics and Neurological Surgery, University of California, San Francisco, San Francisco, CA, USA; 8 Department of Pediatrics, Perelman School of Medicine at the University of Pennsylvania, Philadelphia, PA, USA; 9 Lead contact

## Abstract

Neuroblastoma is the most common extracranial solid tumor of childhood. While *MYCN* and mutant anaplastic lymphoma kinase (*ALK*^*F1174L*^) cooperate in tumorigenesis, how ALK contributes to tumor formation remains unclear. Here, we used a human stem cell-based model of neuroblastoma. Mis-expression of *ALK*^*F1174L*^ and *MYCN* resulted in shorter latency compared to *MYCN* alone. *MYCN* tumors resembled adrenergic, while *ALK*/*MYCN* tumors resembled mesenchymal, neuroblastoma. Transcriptomic analysis revealed enrichment in focal adhesion signaling, particularly the extracellular matrix genes *POSTN* and *FN1* in *ALK*/*MYCN* tumors. Patients with *ALK*-mutant tumors similarly demonstrated elevated levels of *POSTN* and *FN1*. Knockdown of *POSTN*, but not *FN1*, delayed adhesion and suppressed proliferation of *ALK*/*MYCN* tumors. Furthermore, loss of *POSTN* reduced *ALK*-dependent activation of WNT signaling. Reciprocally, inhibition of the WNT pathway reduced expression of *POSTN* and growth of *ALK*/*MYCN* tumor cells. Thus, *ALK* drives neuroblastoma in part through a feedforward loop between POSTN and WNT signaling.

## In brief

Huang et al. used a human stem cell model to elucidate the mechanism for cooperation between *MYCN* and *ALK*. *ALK* contributes to tumor growth by upregulating the extracellular matrix protein periostin and activating WNT signaling. Periostin and WNT signal through a feedforward loop.

## Graphical Abstract

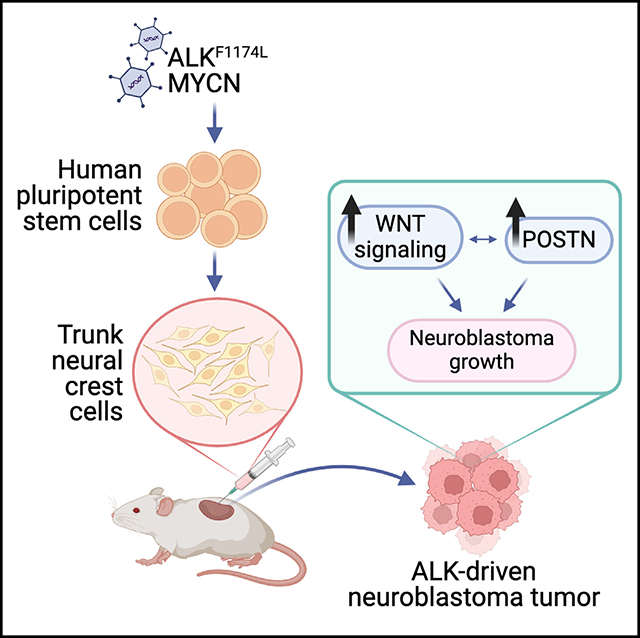

## INTRODUCTION

Neuroblastoma is the most common extracranial solid tumor of childhood and accounts for ~15% of pediatric cancers. Tumors typically arise in the adrenal medulla and less commonly in abdominal, pelvic, or spinal sympathetic ganglia.^1^ Neuroblastoma is composed of two distinct cell identities that represent different states of lineage differentiation. Adrenergic cell states are committed to sympathoadrenal specification, which includes expression of *PHOX2A*, *PHOX2B*, and *GATA2*. In contrast, mesenchymal cell states represent undifferentiated cells related to migrating neural crest and express markers *FN1*, *VIM*, and *SNAI2*.^2,3^ These two cell states co-exist in primary tumors. In cell culture, they are interconvertible, depending on the super-enhancer landscape affecting expression of lineage-defining transcription factors that constitute the core regulatory circuitry of neuroblastoma.

Neuroblastoma likely originates from multipotent neural crest cells (NCCs). NCC subtypes differ depending on where, anatomically, cells dissociate from along the neural tube axis: (anterior) cranial, vagal, trunk, or sacral (posterior).^4^ While NCCs from different regions share similar markers and can differentiate to similar cell types, they also generate distinct cell types and populate different parts of the body. Relevant to neuroblastoma, the adrenal medulla is composed of sympathetic neurons and chromaffin cells. Each of these cell types arises from a common sympathoadrenal progenitor derived from trunk NCCs (tNCCs).^5^ Thus, the tNCC is a likely cell of origin for neuroblastoma.

The most frequently arising recurrent genetic changes in neuroblastoma are amplification of the oncogene *MYCN* and amplification/gain-of-function mutations of *ALK* (anaplastic lymphoma kinase).^6–11^ Mouse and zebrafish models demonstrated that both *MYCN* and activating mutant *ALK*^*F1174L*^ can promote tumorigenesis.^12–15^ However, how *ALK* and *MYCN* conspire to accelerate tumorigenesis remains incompletely understood. Given that human, fish, and mouse cells do not always respond to genetic mutations in the same way,^16,17^ murine and fish models may not fully elucidate mechanisms of *ALK* and *MYCN* cooperation in human tumors. In addition, while human neuroblastoma cell lines can contain both mutant/amplified *ALK* and amplified *MYCN*, these tumor-derived lines also have other mutations that likely impact effects of *ALK* and *MYCN*. Isogenic human stem cell models thus represent a clean system to elucidate the mechanism by which *ALK* and *MYCN* cooperate in high-risk neuroblastoma.

The advent of induced pluripotent stem cell (iPSC) biology enables a renewable source of human iPSC-based tNCCs, providing a reliable, representative system to study neuroblastoma. Human pluripotent stem cell models of neuroblastoma were recently described.^18–20^ These early protocols made NCCs that defaulted to the cranial subtype absent any posteriorizing factors. We previously generated trunk-specific NCCs from human pluripotent stem cells using the caudalizing factor retinoic acid.^21^ Here, we used this protocol to generate tumors driven by *MYCN* with or without *ALK*^*F1174L*^ that resembled neuroblastoma. We leveraged this model to uncover mechanisms by which *ALK* and *MYCN* cooperate in the pathogenesis of neuroblastoma.

## RESULTS

### *MYCN* blocks differentiation of human iPSCs toward tNCCs

Because the differentiation of iPSCs to tNCCs is not a terminal differentiation, the reproducibility of our experiments was best when we stably expressed transgenes at the iPSC stage. Lentiviral constructs expressing constitutive FLAG-tagged *MYCN* were generated and introduced into iPSCs, with expression maintained during NCC differentiation (Figures S1A and S1B). Constitutive expression of MYCN at the iPSC stage interfered with NCC differentiation. Specifically, mRNA and protein expression of AP2A, NGFR/p75, B3GAT1/HNK1, and SOX9 was reduced in cells transduced with *MYCN* (Figures S1C and S1D). Thus, transduction of *MYCN* at the iPSC stage inhibited differentiation toward tNCCs, limiting the ability to model neuroblastoma.

### Doxycycline-inducible *MYCN* facilitated differentiation toward tNCCs, driving neuroblastoma

**Figure 1.**
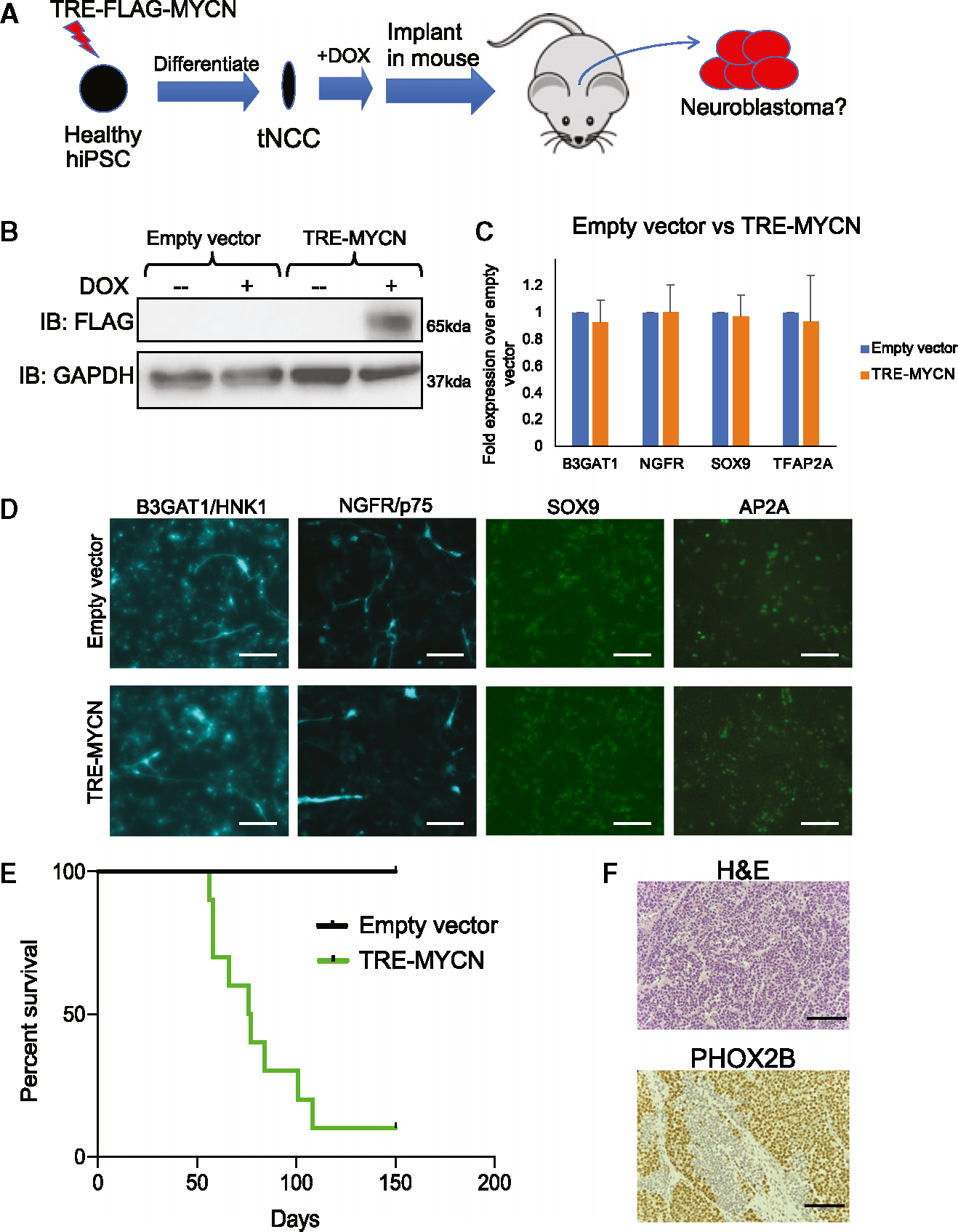
*MYCN* mis-expression drives neuroblastoma formation *in vivo*. (A) Schematic showing a doxycycline (dox)-inducible FLAG-MYCN (TRE-*MYCN*) lentivirus vector is transduced into human iPSCs, differentiated toward tNCCs, treated with dox, and implanted orthotopically into immunocompromised NSG mice. (B) Western blot showing FLAG-tagged *MYCN* expression can by modulated with addition of 0.1 μg/mL dox for 24 h. (C and D) Empty vector and TRE-MYCN iPSCs were differentiated toward tNCCs and analyzed for expression of *B3GAT1*/HNK1, *NGFR*/p75, *SOX9*, and *TFAP2A*/AP2A via (C) RT-qPCR (n = 3, error bars represent standard error of mean) and (D) immunofluorescence. Scale bar: 90 μm. (E) Kaplan-Meier survival curve of mice injected with empty vector or TRE-MYCN tNCCs and fed with dox chow (n = 10). p < 0.05 (log-rank test). (F) Immunohistochemical staining for H&E and PHOX2B in TRE-*MYCN* tumors. Scale bar: 190 μm. See also Figures S1 and S2.

To circumvent differentiation blockade by *MYCN*, we generated doxycycline-inducible tetracycline responsive element-*MYCN* (TRE-*MYCN*) WTC11 iPSCs to induce expression of *MYCN* after differentiation to tNCCs (Figures 1A and 1B). Differentiation of TRE-*MYCN* iPSCs to tNCCs produced cells with levels of NCC markers similar to those in empty vector control tNCCs (Figures 1C and 1D). Orthotopic transplantation of TRE-*MYCN* tNCCs generated tumors 56–108 days after injection at 90% penetrance (Figure 1E). We repeated this with a female iPSC line (1323),^22^ also observing tumors between 53 and 117 days post-injection at 80% penetrance (Figure S2A). Histological analysis showed small-round-blue-cell morphology and positive staining for PHOX2B (Figures 1F and S2B). Therefore, induction of MYCN at tNCCs generates tumors resembling neuroblastoma.

### *MYCN* human stem cell-derived tumors resemble human adrenergic neuroblastoma

**Figure 2.**
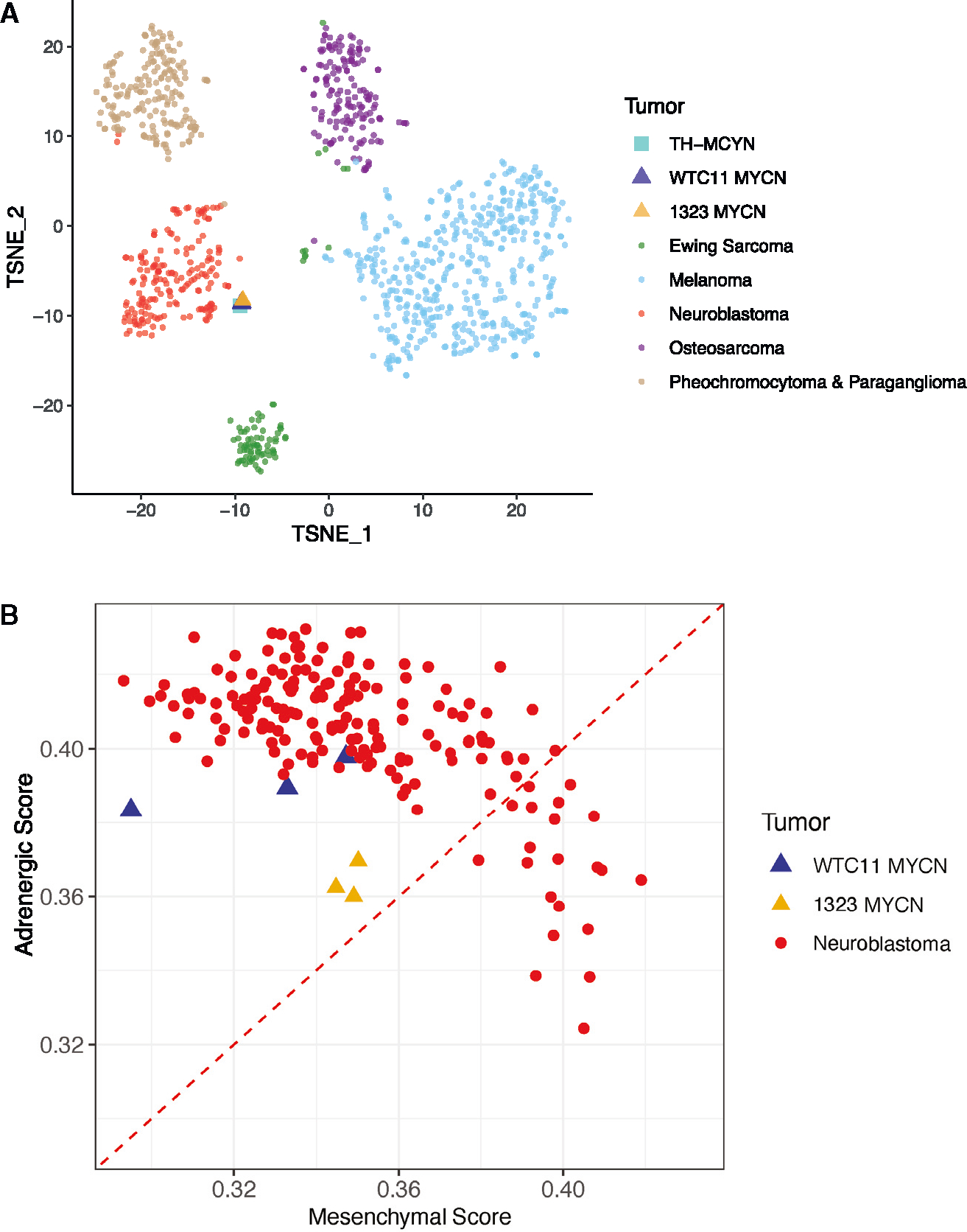
*MYCN*-driven iPSC-derived tNCC tumors resemble adrenergic neuroblastoma. (A) RNA was extracted from tumors derived fromWTC11 and 1323 TRE-*MYCN* tNCCs and analyzed by RNA-seq. Transcriptome profiles of human WTC11 and 1323 tumors and mouse TH-*MYCN* tumors were compared against human neural crest-derived tumors (Ewing sarcoma, melanoma, neuroblastoma, osteosarcoma, pheochromocytoma, and paraganglioma). (B) Transcriptomes of WTC11 and 1323 TRE-*MYCN* tumors were compared against neuroblastoma tumors based on the adrenergic score vs. mesenchymal score. See also Figures S1 and S2.

RNA sequencing (RNA-seq) analysis showed that iPSC-derived tumors (from both WTC11 and 1323 lines) matched neuroblastoma more closely than neural-crest-derived small-round-blue-cell tumors including pheochromocytoma, melanoma, Ewing sarcoma, and osteosarcoma (Figure 2A). TRE-*MYCN* iPSC-derived neuroblastoma tumors aligned more closely with adrenergic than mesenchymal neuroblastoma (Figure 2B), an observation consistent with *MYCN*-amplified human neuroblastoma cell lines.^23^

### Active *ALK* cooperates with MYCN to inhibit apoptosis and accelerate neuroblastoma growth

**Figure 3.**
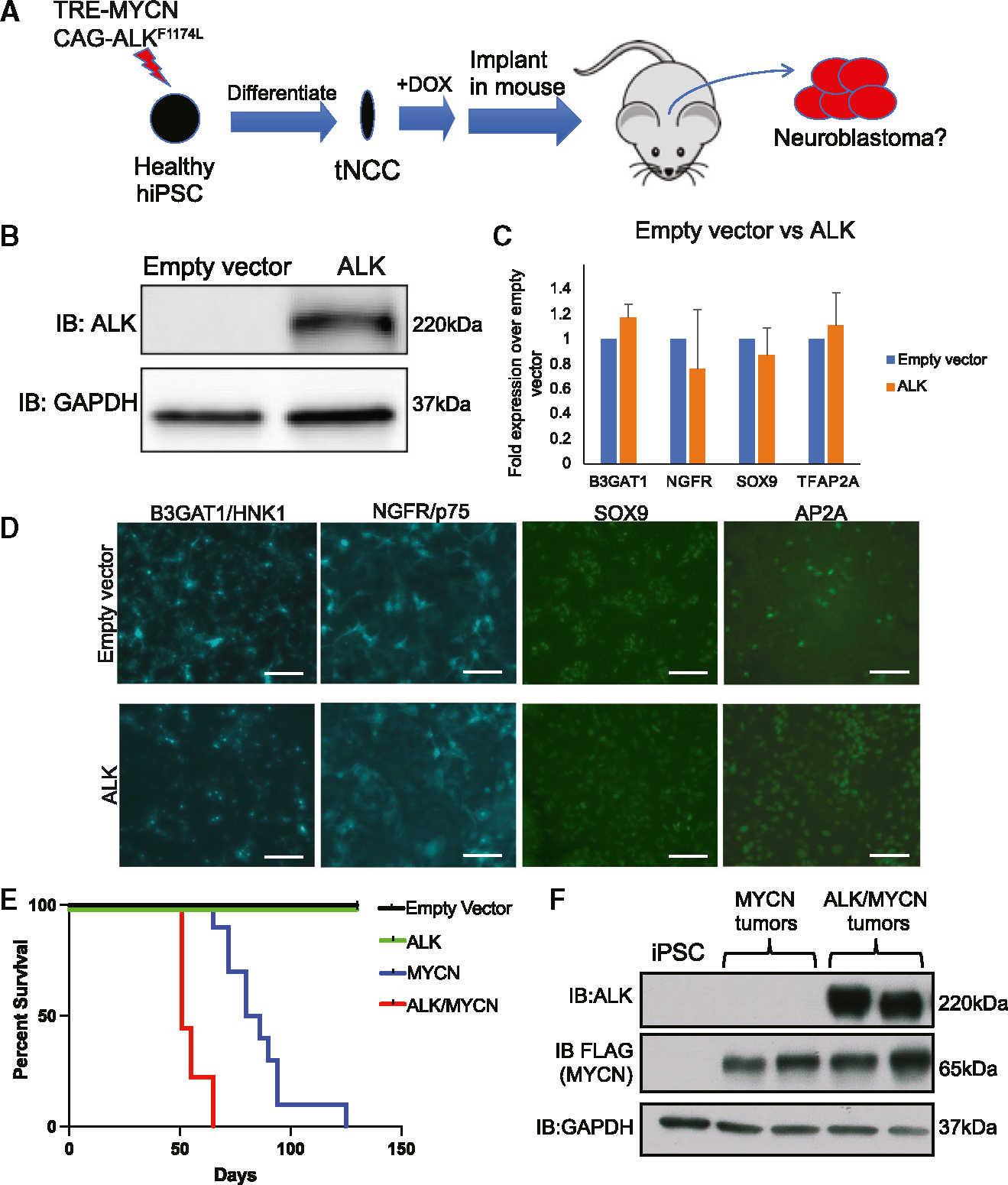
*ALK*^*F1174L*^ cooperates with *MYCN* to accelerate tumorigenesis. (A) Schematic of iPSCs transduced with inducible *MYCN* expression and/or *ALK*^*F1174L*^ and implanted in renal capsules of NSG mice. (B) Western blot validating expression of ALK in*ALK*^*F1174L*^ iPSCs. (C and D) Empty vector and ALK iPSCs were differentiated toward tNCCs and analyzed for expression of *B3GAT1*/HNK1, *NGFR*/p75, *SOX9*, and *TFAP2A*/AP2A via (C) RT-qPCR (n = 3, error bars represent standard error of mean) and (D) immunofluorescence. Scale bar: 90 μm. (E) Kaplan-Meier survival curve of mice injected withempty vector (black), ALK tNCCs (green), MYCN tNCCs (blue), or MYCN/ALK tNCCs (red) (n = 10 per group). p < 0.05 between MYCN and MYCN/ALK groups by log-rank test. (F) Western blot analysis validating expression of ALK and MYCN in two separate MYCN and ALK/MYCN tumors. See also Figures S3 and S4.

We next asked whether activated *ALK* could cooperate with *MYCN* in our human iPSC-based model. We first demonstrated that constitutive expression of ALK^F1174L^ did not affect expression of pluripotency markers nor differentiation from iPSCs to tNCCs (Figures S3 and 3). iPSCs transduced with empty vector and *ALK*^*F1174L*^ were differentiated toward tNCCs and analyzed for expression of NCC markers via RT-qPCR and immunofluorescence (Figure 3B). Levels of AP2A, NGFR/p75, B3GAT1/HNK1, and SOX9 were not impacted by expression of ALK^F1174L^ (Figures 3C and 3D). This allowed us to generate stable *ALK*^*F1174L*^ iPSCs with or without TRE-*MYCN*, resulting in an allelic series of empty vector, *ALK*^*F1174L*^, TRE-*MYCN*, and TRE-*MYCN*/*ALK*^*F1174L*^ iPSCs. Each of the four transgenic iPSCs were differentiated toward tNCCs and implanted orthotopically into NSG mice. *ALK*^*F1174L*^ was not sufficient to drive tumors (Figure 3E). *ALK*^*F1174L*^ cooperated with *MYCN* to shorten the latency of tumor formation (Figure 3E) compared to *MYCN* alone (51–65 vs. 65–125 days, respectively). Western blot analysis confirmed the expression of MYCN and ALK in appropriate tumors (Figure 3F). While we did not observe differences in Ki67 (proliferation), CD34 (angiogenesis), or expression of PHOX2B (adrenergic marker), we found higher expression of vimentin (mesenchymal marker) and lower expression of cleaved caspase-3 (apoptosis) in *ALK*/*MYCN* tumors (Figure S4). Thus, *ALK*^*F1174L*^ cooperated with *MYCN* to reduce apoptosis and accelerate tumor growth in the human stem cell model.

### Activated *ALK* promotes expression of mesenchymal markers and focal adhesion signaling

**Figure 4.**
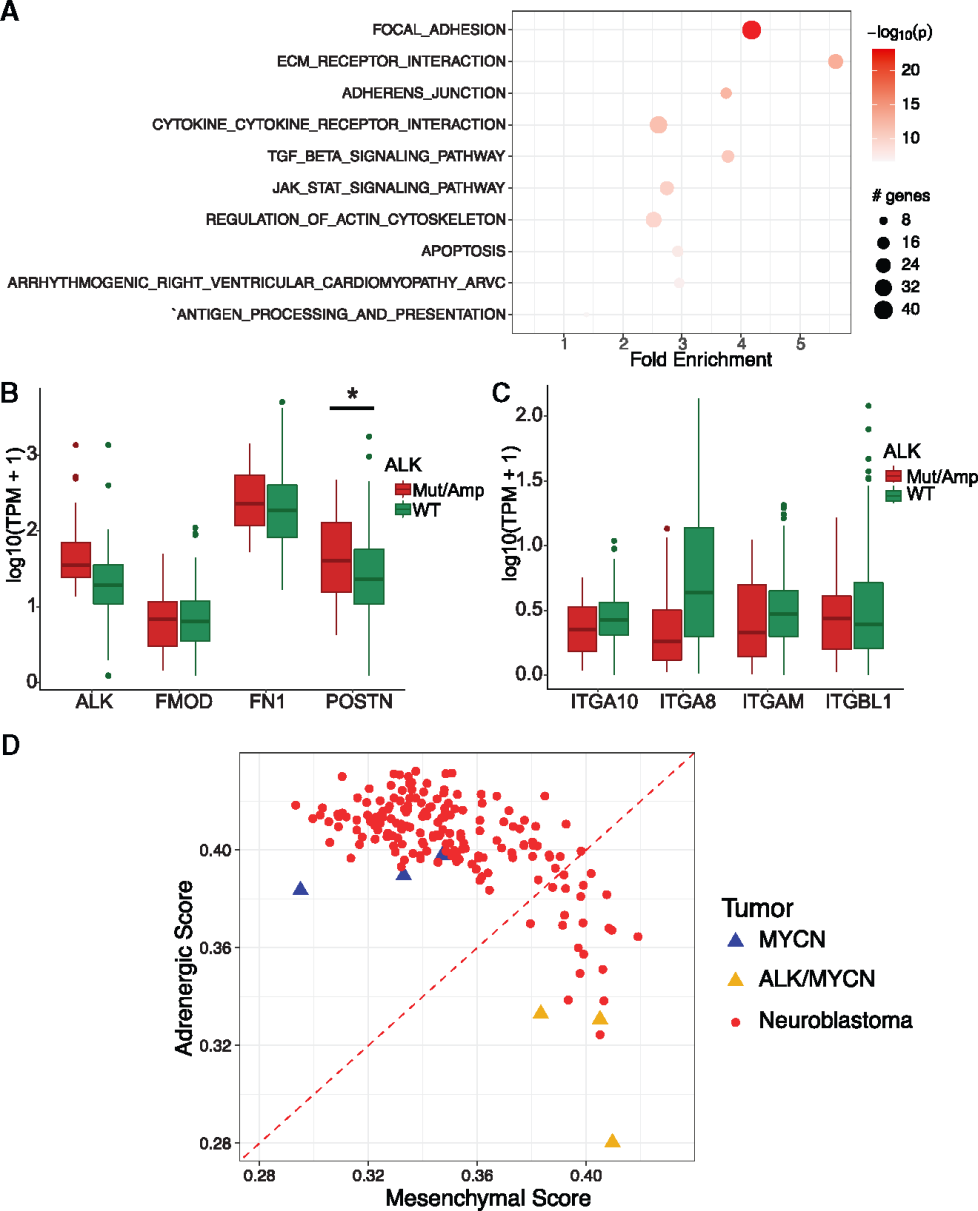
ALK-driven tumors upregulate expression of ECM proteins and resemble mesenchymal neuroblastoma. (A) Gene Ontology analysis showing KEGG database pathways enriched in ALK/MYCN tumors compared to MYCN alone. (B and C) Comparison between *ALK* amplified/mutant (red) against *ALK* wild type (green) for expression of (B) ECM genes FMOD, *FN1*, and *POSTN* and (C) integrin genes *ITGA10*, *ITGA8*, *ITGAM*, and *ITGBL1*. Data were obtained from the TARGET and GMKF databases. Error bars represent 95% confidence interval. There were 301 *ALK* wild-type samples and 24 *ALK*-amplified/-mutant samples, and statistical differences between the two groups was calculated using Wilcoxon rank-sum test. *p < 0.05. (D) Transcriptomes of MYCN and ALK/MYCN tumors were compared against neuroblastoma tumors based on the adrenergic score vs. mesenchymal score. See also Table S1 and Figure S5.

To explore how *ALK*^*F1174L*^ cooperates with *MYCN* to accelerate tumor growth, we performed RNA-seq on the *MYCN* and *ALK*^*F1174L*^/*MYCN* tumors (hereafter referred to as “*ALK*/*MYCN*”). Interestingly, focal adhesion, extracellular matrix (ECM) receptor interaction, and regulation of actin cytoskeleton represented top pathways enriched in *ALK*/*MYCN* tumors (Figure 4A). Since integrins and their ligands, ECM are involved in these processes, we examined the most upregulated integrins and ECMs in *ALK*/*MYCN* tumors: *ITGA8*, *ITGA10*, *ITGBL1*, *ITGAM*, *FMOD*, *FN1*, and *POSTN* (Table S1). We next examined human neuroblastoma tumors based on *ALK* status (wild type, amplified/mutated). *FN1* and *POSTN*, but not *FMOD*, showed increased expression in *ALK*-mutant and -amplified tumors (Figure 4B). In contrast, none of the integrin genes were upregulated in the amplified/mutant *ALK* compared to wild-type ALK (Figure 4C). We then examined how expression of *POSTN* and *FN1* impacted patient outcome. Using the R2 database, we analyzed the Maris and Versteeg cohort. In both populations, tumors with higher expression of *POSTN* had worse outcomes than those with lower levels (Figure S5). Since expression of *FN1* has been linked to the mesenchymal state of neuroblastoma,^2,24^ we analyzed the transcriptome of the human stem cell-derived *ALK*/*MYCN* tumors. Indeed, these tumors had a stronger mesenchymal signature compared to *MYCN* tumors, supporting our immunohistochemistry results showing higher levels of vimentin expression in *ALK*/*MYCN* tumors (Figures S4 and 4D).

### *ALK* tumors depend on *POSTN* for adhesion, migration, and growth

**Figure 5.**
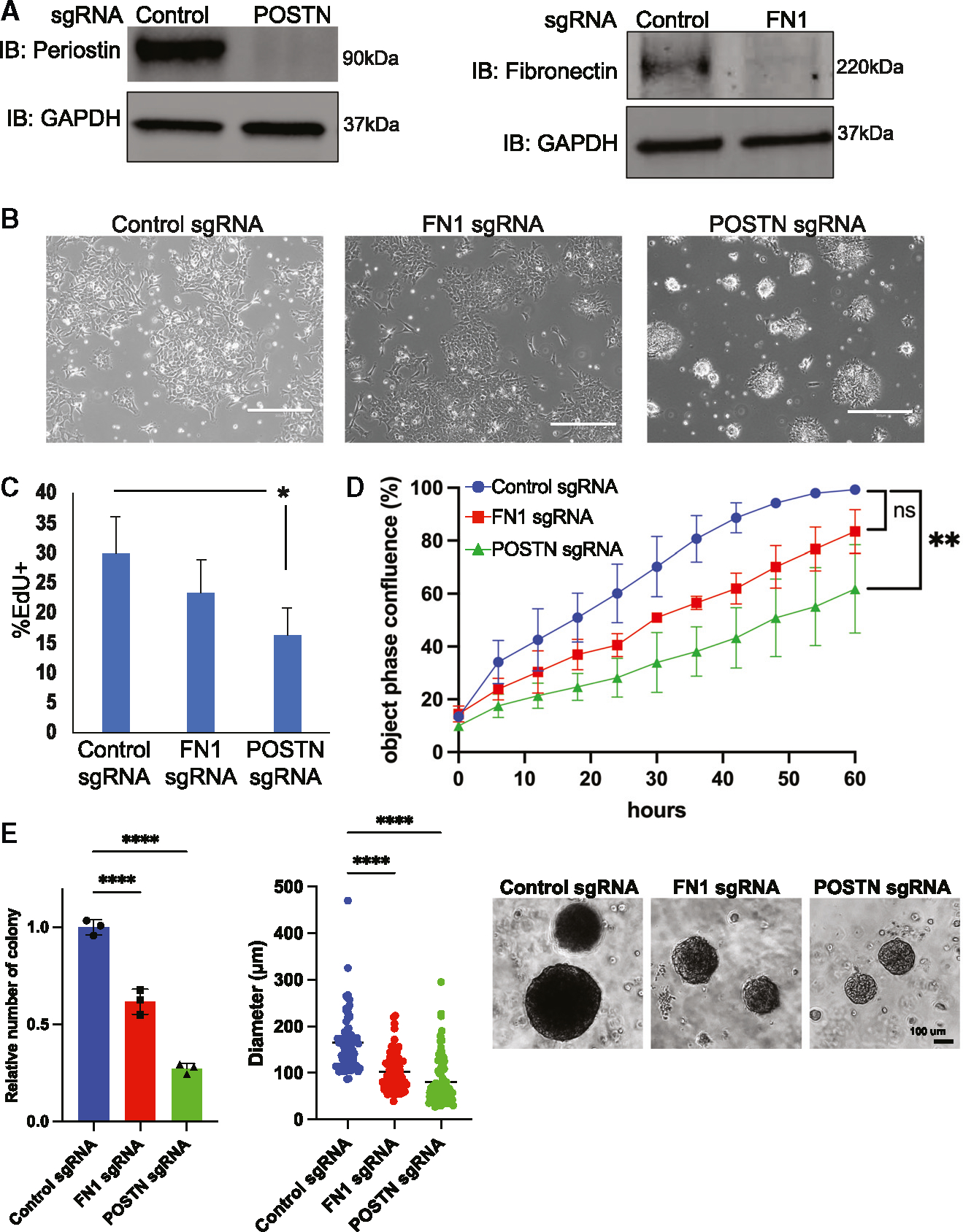
*POSTN*, but not *FN1*, is required for *ALK*/*MYCN* tumor cell adhesion and growth. (A) *ALK*/*MYCN* tumor lines were transduced with dCas9-BFP-KRAB (CRISPR interference [CRISPRi]) and either control sgRNA, *FN1* sgRNA, or *POSTN* sgRNA. Knockdown of *FN1* and *POSTN* was validated by western blot. (B) *ALK*/*MYCN* tumor cells with control, *FN1*, and *POSTN* sgRNAs were plated for 24 h on uncoated tissue culture-treated dishes. Scale bar = 380 μm (C) *ALK*/*MYCN* tumor cells with control, *FN1*, and *POSTN* sgRNAs were treated with 10 μM EdU for 1 h and analyzed by flow cytometry for the percentage of cells that stain positive for EdU. Bar graph shows the average of EdU+ cells in three independent experiments. Error bars represent standard error mean. *p < 0.05 (D) Phase objective confluence calculated by IncuCyte for *ALK*/*MYCN* tumor cells with control, *FN1*, or *POSTN* sgRNA over 60 h. Each datapoint represents mean confluence ± standard deviation (n = 4 for each tumor cell line. **p < 0.01, unpaired t test). (E) ALK/MYCN tumor cells with control, FN1, and POSTN sgRNAs were cultured in soft agar for 3 weeks. Bars represent the mean ± SD (n = 3 for each tumor cell line); ****p < 0.0001. The scale bar shows 100 μm. See also Figure S6.

To assess functional roles for *FN1* and *POSTN* in tumor growth, we knocked down each via CRISPR interference^25^ in *ALK*/*MYCN* tumor lines (Figure 5A). Using the pooled knockdown population, we first evaluated how loss of *POSTN* and *FN1* affected cell adhesion. Twenty-four hours after plating, cells that had loss of *POSTN*, but not *FN1*, exhibited a demonstrable reduction in adhesion (Figure 5B). We then tested whether *POSTN* and *FN1* impacted cell migration in a wound-healing assay. Knockdown of *POSTN*, but not *FN1*, significantly slows the migration rate of *ALK*/*MYCN* tumor cells in filling the wounded area (Figure S6). Next, we tested the impact of *POSTN* and *FN1* in proliferation. Knockdown of *POSTN* decreased EdU incorporation into cells significantly, while knockdown of *FN1* did not (Figure 5C). Similarly, IncuCyte analysis of confluency showed that knockdown of *POSTN* decreased proliferation more than knockdown of *FN1* (Figure 5D). Next, we performed a soft agar assay to analyze how *POSTN* and *FN1* affected anchorage-independent growth. Knockdown of either *POSTN* or *FN1* significantly reduced the number of colonies and the size of the colonies (Figure 5E). These assays show that *POSTN* is required for full cell adhesion, migration, and growth in *ALK*-mutant tumors.

### ALK and POSTN are involved in WNT signaling to promote growth

**Figure 6.**
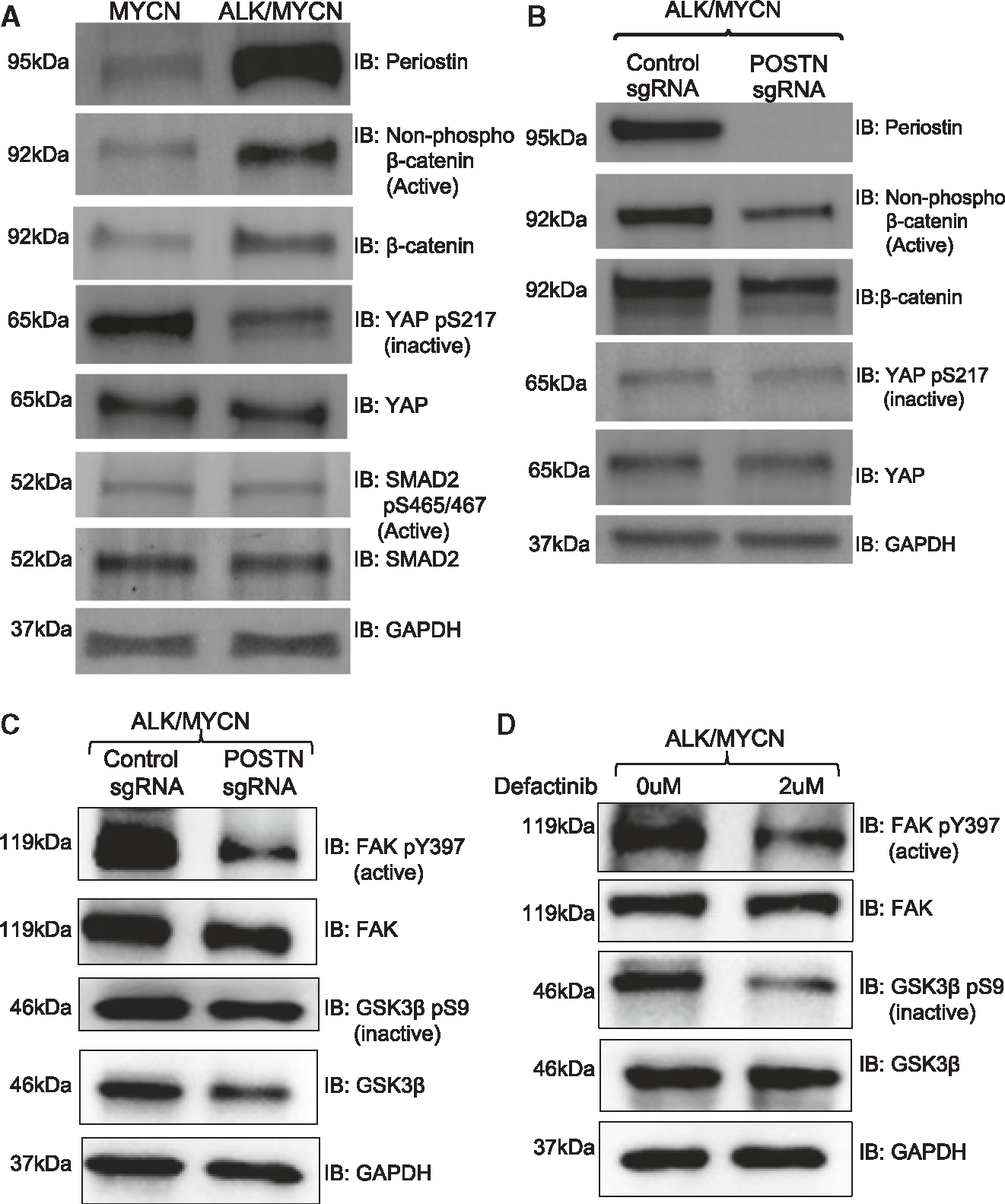
*POSTN* is required for *ALK*-mediated activation of WNT signaling. (A) *MYCN* and *ALK*/*MYCN* tumor lines were analyzed by western blot for expression of POSTN, active form of β-catenin (non-phosphorylated at S33/S37/T41), active SMAD2 (phosphorylated at S465/467), and inactive YAP (phosphorylated at S127) expression and WNT signaling. (B and C) *ALK*/*MYCN* with control sgRNA or *POSTN* sgRNA tumor lines were analyzed by western blot for (B) activation of WNT and YAP signaling and (C) activation of FAK and inhibition of GSK3β. (D) *ALK*/*MYCN* tumor cells were treated with or without the FAK inhibitor defactinib (2 μM) for 48 h and analyzed by western blot for FAK and GSK3β activity. See also Figures S7–S9.

Since periostin has been linked to the activation of transforming growth factor β (TGF-β), YAP, and WNT^26,27^ signaling, we tested whether these pathways were activated by ALK and affected by expression of *POSTN*. Gene Ontology analysis of ALK/MYCN tumors showed a statistically significant enrichment in WNT and TGF-β, but not YAP, pathways (Figure S7). Western blot analysis showed that ALK increased active β-catenin (non-phosphorylated S33/S37/T41) and reduced inactive YAP1 (phosphorylated S127) while not impacting SMAD2 (phosphorylated S465/467) (Figures 6A and S8A). Immunofluorescence staining validated higher levels of YAP in *ALK*/*MYCN* compared to *MYCN* tumors (Figure S9). Thus, ALK induced WNT and YAP signaling but not TGF-β.

Next, we compared the activity levels of WNT and YAP signaling in *ALK*/*MYCN* tumor cells with control single guide RNA (sgRNA) or POSTN sgRNA. Knockdown of *POSTN* did not affect YAP signaling but did reduce the levels of active and total β-catenin (Figures 6B, S8B, and S9), suggesting that POSTN may be important for stabilizing β-catenin. Since GSK3β plays a central role in degrading β-catenin and focal adhesion kinase (FAK) can inhibit GSK3β activity,^28^ we interrogated whether *POSTN* affects activation of FAK and GSK3β. Indeed, knockdown of *POSTN* reduced FAK pY397 (active) and loss of GSK3β pS9 (inactive) (Figure 6C). To evaluate whether FAK activity influences GSK3β, we treated *ALK*/*MYCN* tumor cells with or without the FAK inhibitor defactinib. Inhibition of FAK in *ALK*/*MYCN* tumor cells increased GSK3β activity (Figure 6D). Thus, while *ALK* activated YAP and WNT signaling, periostin only affected the WNT pathway by activating FAK to inhibit GSK3β, which stabilized β-catenin.

**Figure 7.**
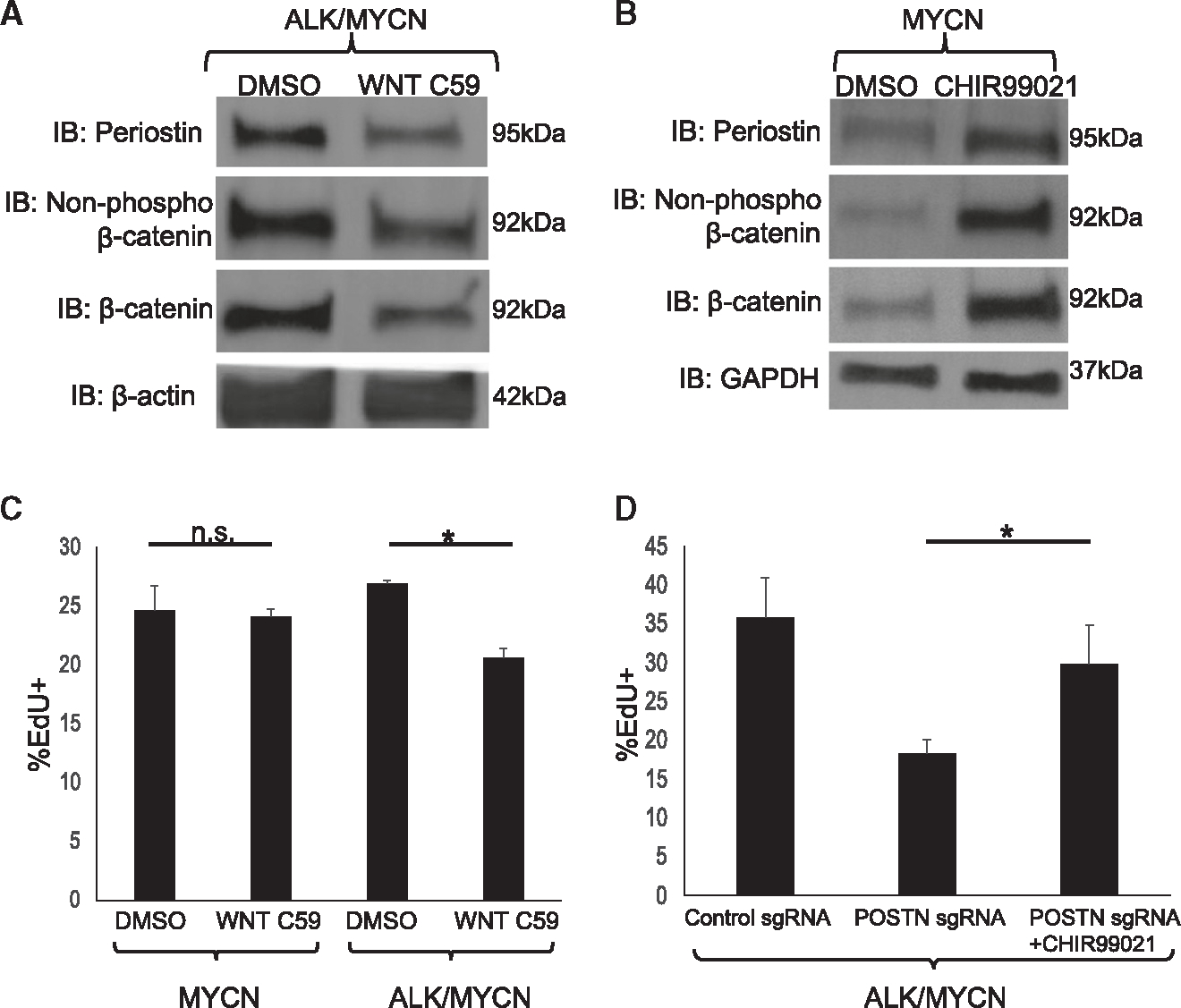
Activation of WNT increases *POSTN* expression and promotes growth of tumor cells. (A) *ALK*/*MYCN* tumor cells are treated with DMSO or 10 μM WNT-C59 for 24 h and analyzed by western blot for *POSTN*, non-phosphorylated β-catenin, and β-catenin. (B) *MYCN* tumor cells were treated with DMSO or 3 μM CHIR99021 for 24 h and analyzed by western blot for *POSTN*, non-phosphorylated β-catenin, and β-catenin. (C) *MYCN* and *ALK*/*MYCN* tumor cells were treated with DMSO or 10 μM WNT C59 for 48 h and analyzed for EdU incorporation. *p < 0.05, n = 3, error bars represent standard error mean. (D) *ALK*/*MYCN* tumor cells with *POSTN* sgRNA were treated with DMSO or 3 μM CHIR99021 for 24 h and analyzed for EdU incorporation. *p < 0.05, n = 3, error bars represent standard error mean. See also Figure S10.

To further investigate how WNT and periostin influenced each other, we first treated *ALK*/*MYCN* tumor cells with the WNT inhibitor WNT-C59 at 10 μM for 48 h. Western blot analysis demonstrated that WNT-C59 reduced expression of *POSTN* (Figures 7A and S10A). Conversely, treatment of *MYCN* tumor cells with the GSK3β inhibitor CHIR99021 (WNT activator) increased periostin (Figures 7B and S10B). Thus, WNT activation is necessary and sufficient for periostin expression, while periostin contributes to activation of WNT signaling.

Next, we examined how changing WNT signaling impacts the growth of *MYCN* and *ALK*/*MYCN* tumor cells. Both cell lines were treated with 10 μM WNT-C59 for 48 h and underwent an EdU incorporation assay. WNT-C59 specifically reduced EdU incorporation in *ALK*/*MYCN* tumor cells but not in *MYCN* tumor cells (Figure 7C). Lastly, we sought to determine whether activation of WNT signaling can rescue the reduced-growth phenotype due to knockdown of *POSTN*. Indeed, treatment with CHIR99021 significantly increased EdU incorporation in *ALK*/*MYCN POSTN* sgRNA tumor cells (Figure 7D). Collectively these data suggest that ALK activates a feedforward loop between WNT and periostin, which are both required for growth in ALK-driven tumors.

## DISCUSSION

Our study demonstrates a link between activation of ALK and tumor growth via increased ECM production and activation of the WNT pathway. Activated *ALK* promoted upregulation of *FN1* and *POSTN* in our human stem cell model and in patients with amplified or mutant *ALK* neuroblastoma (Figures 4B and 4C). Consistent with *FN1* as a marker of mesenchymal neuroblastoma,^2^ *ALK*/*MYCN* tumors were more mesenchymal than *MYCN* tumors (Figure 4D), suggesting that *ALK* can bias neuroblastoma toward the mesenchymal cell state. Periostin, however, appears to be the primary ECM that influences ALK-driven tumor biology. Knockdown of *POSTN* significantly decreased adhesion, migration, and proliferation of cell lines derived from *ALK*/*MYCN* tumors (Figure 5E). *POSTN* signals through a number of downstream pathways.^26^ Surveying these, we found that WNT signaling, but not TGF-β or YAP signaling, required POSTN for full pathway activation (Figure 6). Interestingly, expression of *POSTN* was highly dependent on the activity of WNT. Blocking WNT activity reduced expression of *POSTN*, while activation of WNT increased expression of *POSTN* (Figures 7A and 7B). These data indicate that *ALK* drives tumor biology in part through a feedforward loop between *POSTN* and *WNT* signaling.

Previous human stem cell-based models of neuroblastoma utilized protocols to differentiate human pluripotent stem cells to NCCs.^18,19^ However, these protocols lacked caudalizing factors (e.g., retinoic acid), and resulting NCCs show high expression of *ETS1*, a marker of cranial NCCs.^21,29^ Although neuroblastoma is believed to arise from tNCCs and not cranial NCCs, mis-expression of *MYCN* in NCCs generated by the early protocols produced tumors that shared features of neuroblastoma. We cannot rule out the possibility that the neural crest population was heterogeneous, including tNCCs, or the possibility of NCCs being able to switch subtypes. Trunk and cranial NCCs express similar markers, and few genes can distinguish between these subtypes, highlighting a challenge in using human pluripotent stem cells to generate specific subtypes of NCCs.

While mouse and zebrafish models of cancer have informed early events in tumorigenesis, differences between mouse and human cells, including timelines for development, telomere length, and the efficiency in transformation with similar genes, suggests human stem cell models as complementary to study tumorigenesis. For instance, *MYC* and *RAS* are sufficient to transform mouse fibroblasts but not human fibroblasts.^16,17,30^ *MYCN* is sufficient to transform human neuroepithelial stem cells but insufficient to transform mouse neural stem cells.^31,32^ Thus, human stem cell models potentially provide valuable tools to complement findings from studies of rodent and fish cells.

In our model, *ALK*^*F1174L*^ was unable to transform human tNCCs to neuroblastoma in the absence of *MYCN* (Figure 3E). This is consistent with results in mouse and zebrafish models,^13,15,^ and at odds with results in mouse neural crest progenitor cells^33,34^, and in a genetically engineered mouse model driven by the DBH promoter,^14^ all demonstrating that *ALK*^*F1174L*^ was transforming on its own. However, all studies support the finding that *ALK*^*F1174L*^ accelerates tumorigenesis when combined with *MYCN*.

Our study demonstrates several uses for a human stem cell model of neuroblastoma: (1) evaluating tumorigenic effects of cancer mutations in a human cell system, (2) elucidating mechanisms of cooperation between two tumor promoting genes, and (3) understanding the potential order of mutations. Since human cancer cell lines cannot be “untransformed” to a healthy cell, the ability to reproducibly evaluate cancer drivers in an isogenic series of cell lines is a unique strength for human stem cell models.

### Limitations of the study

Multiple protocols have been published on generating tNCCs and their downstream progenitor, sympathoadrenal cells. It is possible that an alternative protocol may more accurately generate tNCCs and downstream progenitors, resulting in a tumor that better resembles neuroblastoma. As observed by others modeling neuroblastoma, we did not detect gross metastases. This could be due to the protocol used to differentiate the cells, the possibility that micrometastases grow better after the primary tumors are removed, or a result of inducing tumor formation in an NCC that was already post-migratory. Lastly, we tested a single gain-of-function mutation in *ALK*. Our results do not distinguish effects of amplification from mutational activation of *ALK*.

## STAR★METHODS

### RESOURCE AVAILABILITY

#### Lead contact

Further information and requests for resources and reagents should be directed to and will be fulfilled by the lead contact, William A Weiss (waweiss@gmail.com).

#### Materials availability

Plasmids generated in this study will be deposited on Addgene. Cell lines will be available upon request and MTA approval.

#### Data and code availability

- RNAseq analysis of MYCN and ALK/MYCN tumors are available in the Gene Expression Omnibus (GEO) database (GSE228194).
- This paper does not report original code.
- Any additional information required to reanalyze the data reported in this work paper is available from the lead contact upon request.

### EXPERIMENTAL MODEL AND STUDY PARTICIPANT DETAILS

#### Animals

Immunocompromised (NOD-scid IL2Rgamma^null^ or NSG) 6–8 week old female mice used for transplantation were purchased from Jackson Labs. Mice were maintained in the Animal Facility at UCSF. All experiments were performed in accordance with national guidelines and regulations, and with the approval of the IACUC at UCSF. For each *in vivo* experiment, 10^6^ tNCC were resuspended in 10uL of a mixture of 50% GelTrex (Thermo Fisher) and 50% PBS and injected orthotopically into the left renal capsules of mice. Mice were euthanized at endpoint, which was either 2cm length of tumor or 1 year post transplantation. Mice were fed Dox chow immediately after surgical recovery.

#### iPSC culture

Male WTC11^35^ and Female 1323 iPSC^22^ were maintained on GelTrex (Thermo Fisher) coated 6-well plates in mTeSR1 media (Stemcell Technologies) in a humidified 37°C incubator with 5% CO_2_. Cells were passaged every 4–5 days using Accutase (Innovative Cell Technologies) (and plated at 20–30,000 cells/cm^2^) mTeSR1 with 2uM of ROCK inhibitor Thiazovivin (Stemcell Technologies). All experiments using iPSC in the William A Weiss lab (UCSF) were approved by Human Gamete, Embryo and Stem Cell Research Committee of the UCSF Stem Cell Research Oversight Committee.

#### Differentiation of iPSC to tNCC

Differentiation from iPSC to tNCC was performed as previously described with one modification.^21^ Retinoic Acid (Sigma Aldrich) added starting at Day 3 was used at a final concentration of 10uM.

#### tNCC derived tumor lines

Tumor tissue from mice were physically cut with scalpels into as small pieces as possible and dissociated with Accutase at 37°C for 10 min. Cells were then plated in 6-well plates in a modified version of NBE media^40^ consisting of Neurobasal media (Thermo Fisher), 1x Glutamax (Thermo Fisher), 1x N2 supplement (Thermo Fisher), 1x B27 without vitamin A supplement (Thermo Fisher), human recombinant FGF2 and EGF (both from Peprotech at 20 ng/mL). Doxycycline (Sigma Aldrich) was added at 1ug/mL to maintain expression of inducible *MYCN*. Media is changed every 2–3 days and tumor cells were passaged using Accutase.

### METHOD DETAILS

#### Plasmids

We cloned empty vector, doxycycline-inducible MYCN, constitutive MYCN, constitutive ALK^F1174L,^ and constitutive dCas9-BFP-KRAB^41^ in lentiviral plasmids (pCDH backbone). Doxycycline-inducible MYCN plasmid consisted of the following: UCOE-TRE3G-3xFLAG-MYCN-P2A-mScarlet, and CAG-rtta3G-P2A-Luciferase-T2A-Blasticidin. Constitutive ALK plasmid consisted of the following: CAG-ALK^F1174L^ and EF1a-puromycin. Constitutive MYCN plasmid is the same used in a previous study.^32^

For plasmids expressing sgRNA against FN1, POSTN and non-targeting control, we cloned a lentivirus plasmid consisting of U6-sgRNA (from the PX330 plasmid^42^) and CAG-puromycin-P2A-BFP. To clone each sgRNA into the plasmid, primers (Integrated DNA Technologies) for each sgRNA were designed with a forward primer 5′-CACC[sgRNA sequence]-3′ and a reverse primer of 5′-AAAC[reverse complement of sgRNA sequence]-3’. If the 20 nucleotide sgRNA sequence doesn’t start with a “G”, then a “G” was added 5′ to the sgRNA sequence and is included in the reverse complement sequence. Both primers (100μM) are annealed with T4 PNK (NEB) in T4 ligation buffer (NEB) in 10uL total volume using the following protocol: 37C (30 min), 95C (5min), decrease 5C per minute until 25C. Primers mixes were then diluted 1:250 in water and added 1uL of primer dilution to 100ng of the backbone, 2uL of fast digest buffer (Thermo Fisher), 1uL *BbsI* (NEB), 0.5 T7 ligase (NEB), 0.5mM DTT (Thermo Fisher), 0.5mM ATP (NEB) and volume raised to 20uL. The ligation and digestion mixture was run for 6 cycles of a two step process including 37C for 5 min and 23C for 5 min 2uL of ligation and digestion product was transformed in 20uL of Stbl3 competent cells (Thermo Fisher) on LB agar plates with ampicillin. Bacteria colonies were picked and grown in LB Broth with ampicillin overnight. DNA was extracted using a miniprep kit (Zymo Research) and sequence verified with a U6 sequencing primer (5′GACTATCATATGCTTACCGT-3′) at Azenta/Genewiz.

#### Antibodies

Primary antibodies for immunofluorescence were obtained commercially for HNK1 (Sigma Aldrich, 1:500), p75 (Advance Targeting Systems, 1:250), SOX9 (Cell Signaling Technology, 1; 100), AP2A (Thermo Fisher, 1:100), OCT4 (Santa Cruz, 1:500), NANOG (Thermo Fisher, 1:250), SOX2 (Thermo Fisher, 1:500), YAP (Santa Cruz, 1:100), β-catenin (Cell Signaling Technology, 1:300), non-phosphorylated β-catenin (Cell Signaling Technology, 1:300). Secondary antibodies were in the Alexa Fluor spectrum obtained from Thermo Fisher and used at 1:500. Primary antibodies for immunohistochemistry were purchased for PHOX2B (Abcam, 1:100), Ki67 (Thermo Fisher, 1:100), CD34 (Millipore, 1:800), VIM (Cell Signaling Technology, 1:100), cleaved caspase 3 (Cell Signaling Technology, 1:100). Primary antibodies for western blot were obtained commercially for POSTN (Thermo Fisher, 1:1000), FN1 (Cell Signaling Technology, 1:1000), GAPDH (Sigma Aldrich, 1:10000), FLAG (Sigma Aldrich, 1:2000), ALK (Cell Signaling Technology, 1:1000), a-tubulin (Cell Signaling Technologies, 1:1000), FAK (Abcam, 1:1000), pFAK Y397 (Abcam, 1:1000), GSK3β (Cell Signaling Technology, 1:1000), pGSK3β S9 (Cell Signaling Technology, 1:1000), β-catenin (Cell Signaling Technology, 1:1000), non-phosphorylated β-catenin (Cell Signaling Technology, 1:1000), SMAD2 (Cell Signaling Technology, 1:1000), pSMAD2/3 (Cell Signaling Technology, 1:1000), YAP (Santa Cruz, 1:1000), pYAP (Cell Signaling Technology, 1:1000). Secondary mouse and rabbit antibodies for western blots were obtained from Bio-Rad and used at 1:5000.

#### RT-qPCR

RNA was extracted using a Quick-RNA Miniprep kit (Zymo Research). 500ng of RNA was converted to cDNA using VILO Superscript (Thermo Fisher) or the high-capacity cDNA Reverse Transcription Kit (Applied Biosystems) in a 20uL final volume and the following settings: 25°C for 10min, 42°C for 60 min and 85°C for 5 min cDNA was then diluted in 80uL of water and qPCR was performed in a 384 well plate using SYBR green (Bio-Rad) on an CFX384 machine (Bio-Rad) with the following settings: 95°C for 1 min, 40 cycles (95°C for 3 s and 60°C for 1 min). Each qPCR reaction occurred in final volume of 10uL containing 5uL SYBR mastermix, 5uM of each forward and reverse primer, and 0.4uL of the cDNA. Gene expression was normalized to GAPDH expression and represented as fold increase over the control cell lines.

#### Immunohistochemistry

Extracted tumors from mice were fixed with 10% neutral buffered formalin (Sigma) for 24 h and changed to 70% ethanol (Sigma). Tumors were paraffin-embedded and sectioned by the Brain Tumor Research Center at UCSF and CHLA. For IHC, slides were deparaffinized, and antigen retrieval was performed using a pressure cooker. The Mouse on Mouse (M.O.M.) basic kit (Vector laboratories) was used for masking endogenous mouse antigen. The VECTASTAIN ABC kit (Vector laboratories) was used for signal detection. Images were taken using the Revolve microscope (ECHO).

#### Immunofluorescence

Cells were plated at 150,000 per well on 12mm cover glasses (Fisher Scientific) in 24 well plates and cultured for 48 h. Cells were then washed with 1xDPBS (Thermo Fisher), fixed in 4% formaldehyde solution (Sigma Aldrich) for 10 min, permeabilized with 0.3% Triton X-100 (Sigma) in 1x DPBS for 30 min at 37°C, and blocked for 1 h at room temperature with 1% BSA (Sigma) in 1x DPBS. Cover glasses were then incubated overnight at 4°C with primary antibodies diluted in 1% BSA in 1x DPBS, washed 3 times in 1x DPBS, and incubated with secondary antibodies at room temperature for 1 h diluted in 1x DPBS. Coverslips are mounted with Fluoromount-G mounting medium with DAPI (Thermo Fisher) on single frosted micro slides (Corning). Fluorescent images were taken using an Echo Revolve (for NCC markers) at 20x and Nikon confocal at 63x oil immersion for YAP staining.

#### Small molecule inhibitors

WNT-C59 (SelleckChem, 10uM), SB431542 (StemRD, 10uM), Defactinib (SelleckChem, 2uM) and CHIR99021 (Tocris, 3uM), Retinoic Acid (Sigma Aldrich, 10uM), were commercially obtained.

#### Western Blotting

Cells were lysed using RIPA buffer (Thermo Fisher) with HALT protease and phosphatase inhibitor (Thermo Fisher) then sonicated 3 times for 3 s each at an amplitude of 15 s using sonicator (Fisher Scientific). Primary and secondary antibody incubations are performed as recommended diluted in TBST (Bio-Rad). Membranes were developed by ECL (Bio-Rad).

#### Quantification of western blots

Western blot quantification was performed by ImageJ with the gel analysis tool. Lanes of band intensities were plotted as bell curves and the areas under the curve were quantified. Band densities of the target proteins were normalized to their respective loading controls, and relative densities were normalized to the density of the target protein of the control cell line. Quantifications shown is the average of band densities in triplicate and standard deviations.

#### Cell proliferation assays

EdU cell proliferation kit (Thermo Fisher) was used according to manufacturer protocol. For WNT inhibition experiments, cells were treated without or with 10uM WNT-C59 (Selleckchem) for 48 h prior to EdU treatment. For WNT activation experiments, cells were treated without or with 3uM CHIR99021 (Tocris) for 24 h prior to EdU treatment. Cells were then treated with 10uM EdU for 1 h, fixed and undergone Click-iT chemistry to stain for EdU in cells. EdU-treated cells are analyzed through the BD FACS Canto II machine using the BD FACSDiva Software (BD Biosciences).

For growth assays by Incucyte (Sartorius), phase objective confluence is calculated by Incucyte after imaging every 6 h over a total of 60 h. 150,000 cells per well are plated in a 12 well plate. 16 images are taken in different sections of each well to calculate the average confluence.

#### Soft agar colony formation assay

1.2% agarose (Sigma Aldrich) was plated in 12-well plate and allowed to gel at room temperature. Cells were mixed with NBE media in 0.6% agarose and plated on top of the base layer. Cells were incubated for 3 weeks and the ECHO Revolve microscope (ECHO) was used to captured images and to quantify the number of colonies and their sizes.

#### Migration assay

500,000 cells were plated per well in a 24-well plate. After 24 h, the bottom of the wells were scratched with a 200 μL pipette tip. Wells were washed once with 1xDPBS to remove detached cells and cells were fed NBE media. The plate was placed in IncuCyte and images of cells with 10x objective were taken every 2 h for 24 h. The wound area was measured using an ImageJ plugin described previously.^43^ Graphpad Prism was used to plot the area data and perform the t test.

#### RNA-seq sample collection

Total RNA was extracted from flash frozen tissue using the Tissuelyzer (Qiagen) and AllPrep DNA/RNA Mini Kit (Qiagen). Quality of total RNA samples was checked on an Agilent Bioanalyzer 2100 RNA Nano chip (Agilent) and sent to Novogene (https://en.novogene.com/) for library preparation (polyA enrichment) and RNA sequencing (150 base pair Paired End reads, 50 million reads total).

#### RNA sequencing and analysis

RNA-sequencing reads were mapped to the human hg38 primary assembly reference genome using the STAR aligner v2.7.2a.^36^ Gene expression was quantified as transcript per million (TPM) expression values using RSEM v1.3.1^37^ and the gencode v25 gene annotations. T-distributed Stochastic Neighbor Embedding (t-SNE) was performed using the Rtsne R package.^44^ Differential Gene expression analysis was performed using the DESeq2 R package.^38^ Gene set enrichment was determined using the EnrichR R package.^39,45^ Single sample scoring of adrenergic and mesenchymal molecular phenotypes were calculated using the singscore R package^46^ and the adrenergic and mesenchymal gene signatures described by van Groningen et al.^2^ ALK mutations in TARGET and GMKF patients with neuroblastoma were determined using the DNA-seq Alignment and Variant Calling Workflow and the Somatic CNV Calling workflow from OpenPedCan.^47^

#### Datasets

MYCN and ALK^F1174L^ driven iPSC-based-tNCCs are available in the Gene Expression Omnibus (GEO) database (GSE228194). Neural crest derived transcriptomes were used from the Treehouse Childhood Cancer Initiative compendium (GSE129326). Additional Neuroblastoma DNA and RNA sequencing data was retrieved from Therapeutically Applicable Research to Generate Effective Treatments (TARGET) Neuroblastoma dataset (dbGaP ID: phs000467) and the Gabriella Miller Kids First (GMKF) Pediatric Research Program (dbGaP ID: phs001436). Kaplan Meier survival curves for POSTN and FN1 using the Maris and Versteeg cohorts were obtained using the R2 Website (https://hgserver1.amc.nl/cgi-bin/r2/main.cgi?open_page=login).

#### Statistical analysis

For RT-qPCR, migration, EdU incorporation assay, incucyte and soft agar growth assay data points represent the average of at least 3 independent experiments ±standard error of mean and p values were generated by two-tailed t test by Excel or GraphPad Prism. For Kaplan Meier survival curves, p values were calculated by Log rank (Mantel-Cox test) using GraphPad Prism. For all statistical analyses, p value less than 0.05 was interpreted as statistically significant.

## Supplementary Material

1

2

## Figures and Tables

**KEY RESOURCES TABLE:** 

REAGENT or RESOURCE	SOURCE	IDENTIFIER

Antibodies		

β-catenin	Cell Signaling Technology	Cat#: 8480S, RRID: AB_11127855
non-phospho β-catenin	Cell Signaling Technology	Cat#: 19807S, RRID: AB_2650576
FLAG	Sigma Aldrich	Cat#: F1804–5MG, RRID:AB_262044
FN1	Cell Signaling Technology	Cat#: 26836S; RRID:AB_2924220
HNK1	Sigma Aldrich	Cat#: C6680; RRID:AB_1078474
P75	Advanced Targeting Systems	Cat#: AB-N07; RRID:AB_171797
PHOX2B	Abcam	Cat#: ab183741; RRID:AB_2857845
POSTN	Thermo Fisher Scientific	Cat#: PA5–34641, RRID: AB_2551993
Phospho-FAK (Tyr397)	Abcam	Cat#: ab39967, RRID:AB_955850
FAK	Abcam	Cat#: ab40794, RRID:AB_732300
Phospho-GSK-3 (Ser9)	Cell Signaling Technology	Cat#: 5558, RRID: AB_10013750
GSK3B	Cell Signaling Technology	Cat#: 12456, RRID: AB_2636978
Phospho-SMAD2 (Ser465/467)	Cell Signaling Technology	Cat#: 3108; RRID: AB_490941
YAP	Santa Cruz Biotechnology	Cat#: sc-271134, RRID:AB_10612397
Phospho-YAP (Ser217)	Cell Signaling Technology	Cat#: 4911S, RRID: AB_2218913
SMAD2	Cell Signaling Technology	Cat#: 3103; RRID:AB_490816
SOX9	Cell Signaling Technology	Cat# 82630; RRID:AB_2665492
MYCN	Santa Cruz Biotechnology	Cat# 53993; RRID: AB_831602
CD34	Millipore	Cat# CBL496, RRID:AB_93687
VIM	Cell Signaling Technology	Cat# 5741; RRID: AB_10695459
Cleaved Caspase-3	Cell Signaling Technology	Cat# 9661; RRID: AB_2341188
ALK	Cell Signaling Technology	Cat#3633; RRID: AB_11127207
AP2a	Thermo Fisher Scientific	Cat#: PA5–17359; RRID:AB_10982155
OCT4	Thermo Fisher Scientific	Cat# PA5–27438; RRID: AB_2544914
NANOG	Thermo Fisher Scientific	Cat# PA1–097; RRID: AB_2539867
SOX2	Thermo Fisher Scientific	Cat# 14–9811-82; RRID: AB_11219471
Goat anti-rabbit IgG (H + L)-HRP Conjugate	Bio-rad	Cat#: 1706515, RRID:AB_2617112
Goat anti-mouse IgG (H + L)-HRP Conjugate	Bio-rad	Cat#: 1706516, RRID:AB_11125547
Donkey anti-rabbit IgG (H + L) Alexa Fluor 488	Thermo Fisher Scientific	Cat#: SA5–10038; RRID:AB_2556618
Donkey anti-mouse IgG (H + L) AlexaFluor Plus 647	Thermo Fisher Scientific	Cat#: A-31571; RRID: AB_162542
Goat anti-rat IgG (H + L) Alexa Fluor 568	Thermo Fisher Scientific	Cat#: A-11077; RRID: AB_2534121

Chemicals, peptides, and recombinant proteins		

Wnt-C59	Selleckchem	Cat#: S7037
Thiazovivin	Stemcell Technologies	Cat# 72254
SB431542	StemRD	Cat#: SB-050
Defactinib	SelleckChem	Cat#S7654
R3 IGF-1 human	Sigma Aldrich	Cat#: I1146
Geltrex^™^ LDEV-Free, hESC-Qualified, Reduced Growth Factor Basement Membrane Matrix	Thermo Fisher Scientific	Cat#: A1413202
CHIR99021	Tocris	Cat#: 4423
bFGF	Peprotech	Cat# 100–18B
EGF	Peprotech	Cat# 100–15
(+)-sodium L-ascorbate	Sigma Aldrich	Cat#: A4034
10% Neutral Buffered Formalin	Sigma Aldrich	Cat#: R04586–84
10x Tris-buffered saline (TBS)	Bio-Rad	Cat#: 1706435
10x Tris/Glycine/SDS	Bio-Rad	Cat#: 1610772
2-Mercaptoethanol	Thermo Fisher Scientific	Cat#: 21985023
4x Laemmli Sample Buffer	Bio-Rad	Cat#: 1610747
Accutase	Innovative Cell Technologies	Cat#: AT 104–500
B-27 Supplement (50X), without vitamin A	Thermo Fisher Scientific	Cat#: 17504–044
Bovine Serum Albumin (BSA)	Sigma-Aldrich	Cat#: A4612–1G
Clarity Western ECL Substrate	Bio-Rad	Cat#: 1705061
Dulbecco’s phosphate-buffered saline (DPBS), no calcium, no magnesium	Thermo Fisher Scientific	Cat#: 14190144
Ethyl alcohol, Pure	Sigma-Aldrich	Cat#: E7023
EveryBlot Blocking Buffer	Bio-Rad	Cat#: 12010020
Fluoromount-G^™^ Mounting Medium, with DAPI	Thermo Fisher Scientific	Cat#: 00–4959-52
Halt^™^ Protease and Phosphatase Inhibitor Single-Use Cocktail (100X)	Thermo Fisher Scientific	Cat#: 78446
Heregulin β-1	Peprotech	Cat#: 100–03
Mini-PROTEAN TGX Stain-Free Precast Gel	Bio-Rad	Cat#: 4568095
mTeSR^™^1	StemCell technologies	Cat#: 85850
N-2 Supplement (100X)	Thermo Fisher Scientific	Cat#: 17502048
Neurobasal^™^-A Medium	Thermo Fisher Scientific	Cat#: 10888022
Penicillin-Streptomycin (10,000 U/mL)	Thermo Fisher Scientific	Cat#: 15140122
Pierce^™^ RIPA buffer	Thermo Fisher Scientific	Cat#: 89901
Retinoic acid	Sigma-Aldrich	Cat#: R2625
SsoAdvanced Universal SYBR Green Supermix	Bio-Rad	Cat#: 1725271
SuperScript^™^ VILO^™^ Master Mix	Thermo Fisher Scientific	Cat#: 11755250
Trans-Blot Turbo 5x Transfer Buffer	Bio-Rad	Cat#: 10026938
Triton X-100	Sigma-Aldrich	Cat#: X100
ATP (10mM)	New England Biolabs	Cat# P0756L
T7 DNA ligase	New England Biolabs	Cat# M0318L
T4 PNK	New England Biolabs	Cat# M0201L
T4 ligase	New England Biolabs	Cat# M0202L
Bbsi enzyme	New England Biolabs	Cat# R0539L
DTT (1M)	Thermo Fisher Scientific	Cat# P2325
FastDigest Buffer (10x)	Thermo Fisher Scientific	Cat# B64

Critical commercial assays		

Quick RNA Miniprep Kit	Zymo Research	Cat#: 11–328
Pierce^™^ BCA Protein Assay Kit	Thermo Fisher Scientific	Cat#: 23225
Click-iT^™^ Plus EdU Alexa Fluor^™^ 647 Flow Cytometry Assay Kit	Thermo Fisher Scientific	Cat#: C10635
Zyppy Plasmid Miniprep Kit	Zymo Research	Cat# D4037

Deposited data		

MYCN and ALK driven iPSC-based-tNCCs genomic data	GEO: GSE228194	N/A
Treehouse Childhood Cancer Initiative Compendium RNA-seq	GEO: GSE129326	N/A
TARGET Neuroblastoma datasets	dbGaP ID: phs000467	N/A
Gabby Miller Kids First Pediatric Research: Neuroblastoma	dbGaP ID: phs001436	N/A

Experimental models: Cell lines		

WTC11 iPSC	Kreitzer et al., 2013^35^	N/A
1323 iPSC	Matsumoto et al., 2017^22^	N/A

Experimental models: Organisms/strains		

NOD.Cg-Prkdc^scid^ II2rg/Szj	Jackson Labs	Cat#005557; RRID: IMSR_JAX:005557

Oligonucleotides		

FN1 sgRNA (GGCCGGCTGTGCTGCACAGG)	Integrated DNA Technologies	N/A
POSTN sgRNA (GGAGAAAACATGGGTAAAAA)	Integrated DNA Technologies	N/A
B3GAT1/HNK1 qPCR F (TCATCACTGTCTGGCACCAG)	Integrated DNA Technologies	N/A
B3GAT1/HNK1 qPCR R (CAGACGTGCAGTACTCCCTG)	Integrated DNA Technologies	N/A
GAPDHqPCR F (CCATGGGGAAGGTGAAGGTC)	Integrated DNA Technologies	N/A
GAPDH qPCR R (TGAAGGGGTCATTGATGGCA)	Integrated DNA Technologies	N/A
NGFR/p75 qPCR F (GTGAGTGCTGCAAAGCCTG)	Integrated DNA Technologies	N/A
NGFR/p75 qPCR R (CTCACCACGTCGGAGAACG)	Integrated DNA Technologies	N/A
SOX9 qPCR F (GACGCTGGGCAAGCTCT)	Integrated DNA Technologies	N/A
SOX9 qPCR R (GTAATCCGGGTGGTCCTTCT)	Integrated DNA Technologies	N/A
TFAP2A qPCR F (ACTCGGAGACCTCTCGATCC)	Integrated DNA Technologies	N/A
TFAP2A qPCR R (GGACACGGGGCCTTTCTTAA)	Integrated DNA Technologies	N/A

Recombinant DNA		

pCDH-CAG-3xFLAG-MYCN-P2A-mScarlet-T2A-Luciferase	Huang et al., 2016^21^	N/A
pCDH-CAG-ALK^F1174L^-EF1a-puromycin	This paper	N/A
pCDH-CAG-(empty)-EF1a-puromycin	This paper	N/A
pCDH-UCOE-TRE3G-3xFLAG-MYCN- P2A-mScarlet-CAG-rtta3G-P2A-Luciferase-T2A-blasticidin	This paper	N/A
pCDH-UCOE-TRE3G-mScarlet-CAG-rtta3G-P2A-Luciferase-T2A-blasticidin	This paper	N/A
pCDH-U6-control sgRNA-CAG-BFP	This paper	N/A
pCDH-U6-POSTN sgRNA-CAG-BFP	This paper	N/A
pCDH-U6-FN1 sgRNA-CAG-BFP	This paper	N/A
pCDH-CAG-dCas9-BFP-KRAB	This paper	N/A

Software and algorithms		

STAR aligner v2.7.2a	DobinAetal., 2013^36^ https://github.com/alexdobin/STAR/	N/A
RSEM v1.2.1	Li B et al., 2011^37^ https://github.com/deweylab/RSEM	N/A
R	R Core/R Foundation	N/A
RTsne (R Package)	The Comprehensive R Archive Network (CRAN)	N/A
DESeq2 (R Package)	Love MI et al., 2014^38^ Bioconductor	N/A
EnrichR	Chen EYetal., 2013^39^ The Comprehensive R Archive Network (CRAN)	N/A
singscore	Foroutan M etal., 2018^44^ Bioconductor	N/A

## Highlights

- ALK cooperates with MYCN to accelerate tumorigenesis in human neural crest cells
- ALK tumors activate focal adhesion signaling via extracellular matrix proteins
- POSTN and WNT signaling are involved in ALK-mediated tumor growth
