# Supplementary material for: ALK upregulates POSTN and WNT signaling to drive neuroblastoma
Source: Cell Rep. Author manuscript; Available in PMC 2024 May 17. (PMC11101011; doi:10.1016/j.celrep.2024.113927)
Supplement: 1 [file NIHMS1980938-supplement-1.pdf]

Cell Reports, Volume *43*

Supplemental information

ALK upregulates POSTN and WNT signaling to drive neuroblastoma

Miller Huang, Wanqi Fang, Alvin Farrel, Linwei Li, Antonios Chronopoulos, Nicole Nasholm, Bo Cheng, Tina Zheng, Hiroyuki Yoda, Megumi J. Barata, Tania Porras, Matthew L. Miller, Qiqi Zhen, Lisa Ghiglieri, Lauren McHenry, Linyu Wang, Shahab Asgharzadeh, JinSeok Park, W. Clay Gustafson, Katherine K. Matthay, John M. Maris, and William A. Weiss

SUPPLEMENTARY FIGURES

Supplementary Figure 1: Misexpression of *MYCN* at the iPSC stage interferes with tNCC differentiation (A) Schematic showing iPSC were transduced with constitutive FLAG-tagged MYCN, differentiated towards tNCC and analyzed for expression of NCC markers. **(B)** Western blot showing constitutive MYCN expression. **(C-D)** Empty vector and MYCN iPSC were differentiated toward tNCC and analyzed for expression of *B3GAT1*/HNK1, *NGFR*/p75, *SOX9* and *TFAP2A*/AP2A via **(C)** RT-qPCR(n=3, data represent mean $\pm$ SEM), and **(D)** Immunofluorescence. Scale bar = 90um. See also **Figures 1,2.**

Supplementary Figure 2: *MYCN* misexpression drives neuroblastoma formation in a second iPSC-derived tNCC*.* 1323 iPSC were transduced with TRE-MYCN, differentiated toward tNCC and implanted orthotopically into renal capsule of NSG mice (n=10 per group). **(A)** Kaplan Meier curve shows *MYCN* is sufficient to transform 1323 tNCC to generate tumors. p<0.001. **(B)** Histology analysis of 1323 tumors by *(left)* H&E and *(right)* PHOX2B. Scale bar = 90um. See also **Figures 1,2.**

Supplementary Figure 3: ALK does not impact expression of pluripotency markers*.* iPSC transduced with empty vector or ALK^{F1174L} were stained for pluripotency markers by immunofluorescence for OCT4, SOX2 and NANOG. Scale bar = 110um. See also **Figure 3.**

Supplementary Figure 4: Histology of *ALK/MYCN* tumors resemble *MYCN* tumors*.* Tissue from *(left)* WTC11 *MYCN* and *(right) ALK/MYCN* tumors were stained for H&E, PHOX2B, VIM, Ki67, cleaved caspase-3 and CD34. Scale bar = 110um. See also **Figure 3.**

Supplementary Figure 5: Patients with neuroblastoma that have higher expression of *FN1* or *POSTN* have worse outcome. Kaplan Meier survival curves of patients with neuroblastoma from the Maris group and Versteeg group shows worse outcome when the tumors had high expression of either *(top) POSTN* or *(bottom) FN1*. **See also Figure 4.**

Supplementary Figure 6: Knockdown of *POSTN*, but not *FN1*, slow migration in *ALK/MYCN* tumor cells. *ALK/MYCN* tumor cells with control, *FN1* or *POSTN* sgRNA were plated for 24 hours. After the cells were scratched with a pipet tip, the cells were monitored for 8 hours. **(A)** Transmitted light images show the relative confluency of each cell line at *(left)* immediately after the scratch and *(right)* 8 hours after the scratch. **(B)** Bar graph represents the relative wound area remaining 8 hours after the scratch. Statistical analysis was calculated using t-test ** $p<0.01$, n=3, error bars represent 95% confidence interval. **See also Figure 5**

Supplementary Figure 7: Gene ontology analysis of receptor tyrosine kinases show enrichment of TGFβ and WNT signaling in ALK/MYCN tumors. REACTOME, PID, HALLMARK, KEGG and BIOCARTA show enrichment of TGFβ and WNT pathways in ALK/MYCN tumors over MYCN tumors. See also **Figure 6**.

Supplementary Figure 8: ALK activates WNT signaling via upregulation of POSTN. (A-B) Quantitation of western blots from **Figure 6A-B.** Data represent mean $\pm$ SEM., n=3. See also **Figure 6.**

Supplementary Figure 9: ALK activation, but not *POSTN*, promotes YAP signaling. *MYCN*, *ALK/MYCN* control sgRNA, and *ALK/MYCN POSTN* sgRNA tumor cells were fixed and stained for total YAP and DAPI. Nuclear localization of YAP (sign of activation of YAP) was prominent in both ALK/MYCN tumor cell lines compared to MYCN tumor cells. See also **Figure 6**.

Supplementary Figure 10: WNT signaling is necessary and sufficient for expression of *POSTN*. **(A-B)** Quantitation of western blots from **Figure 7A-B.** Data represent mean ± SEM., n=3. See also **Figure 7.**

Figure S1

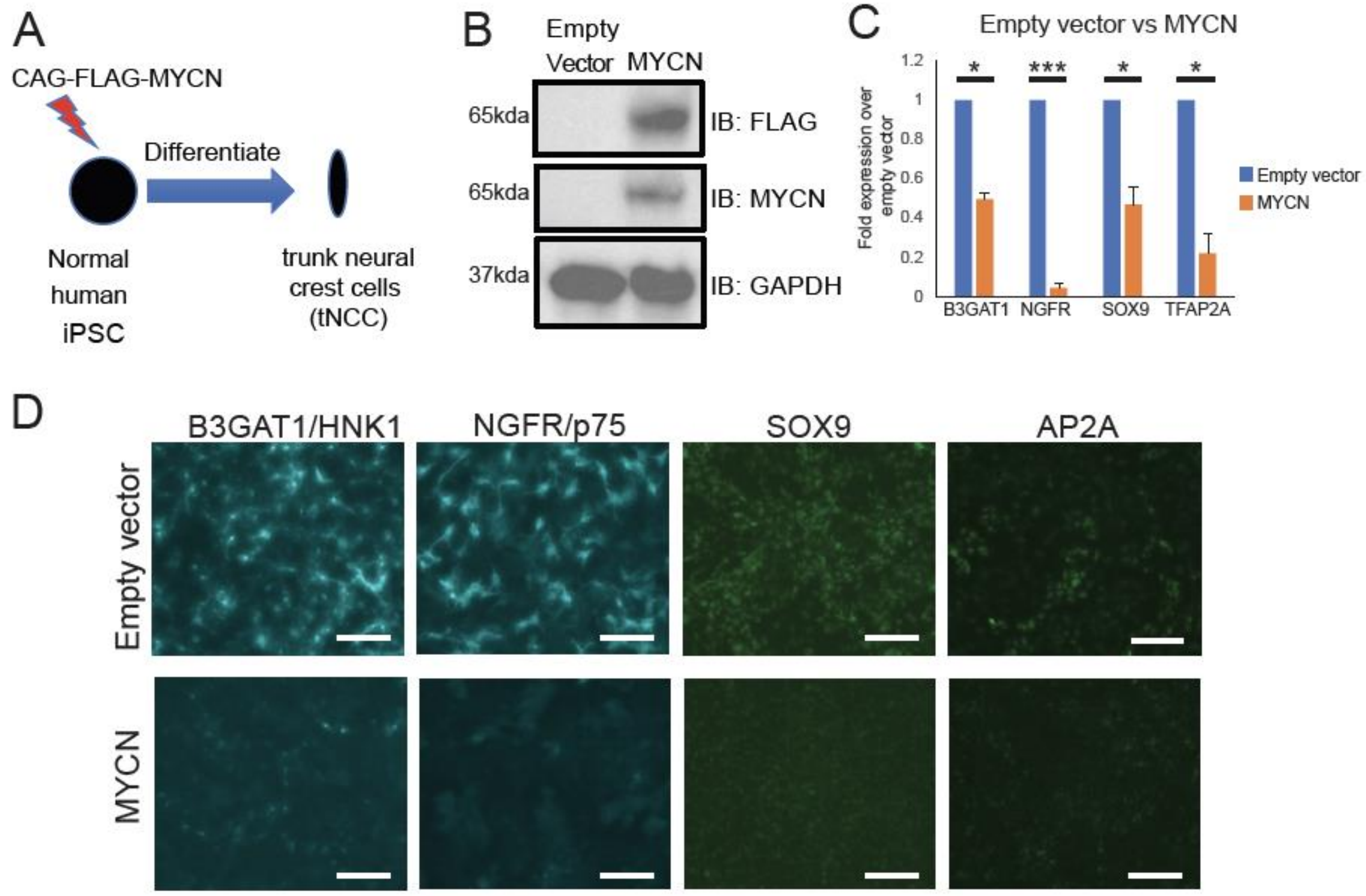
A
CAG-FLAG-MYCN
Differentiate
Normal human iPSC
trunk neural crest cells (tNCC)
B
Empty Vector
MYCN
65kda
65kda
37kda
IB: FLAG
IB: MYCN
IB: GAPDH
C
Empty vector vs MYCN
Fold expression over empty vector
1.2
1
0.8
0.6
0.4
0.2
0
*
***
*
*
B3GAT1
NGFR
SOX9
TFAP2A
Empty vector
MYCN
D
B3GAT1/HNK1
NGFR/p75
SOX9
AP2A
Empty vector
MYCN

Figure S2

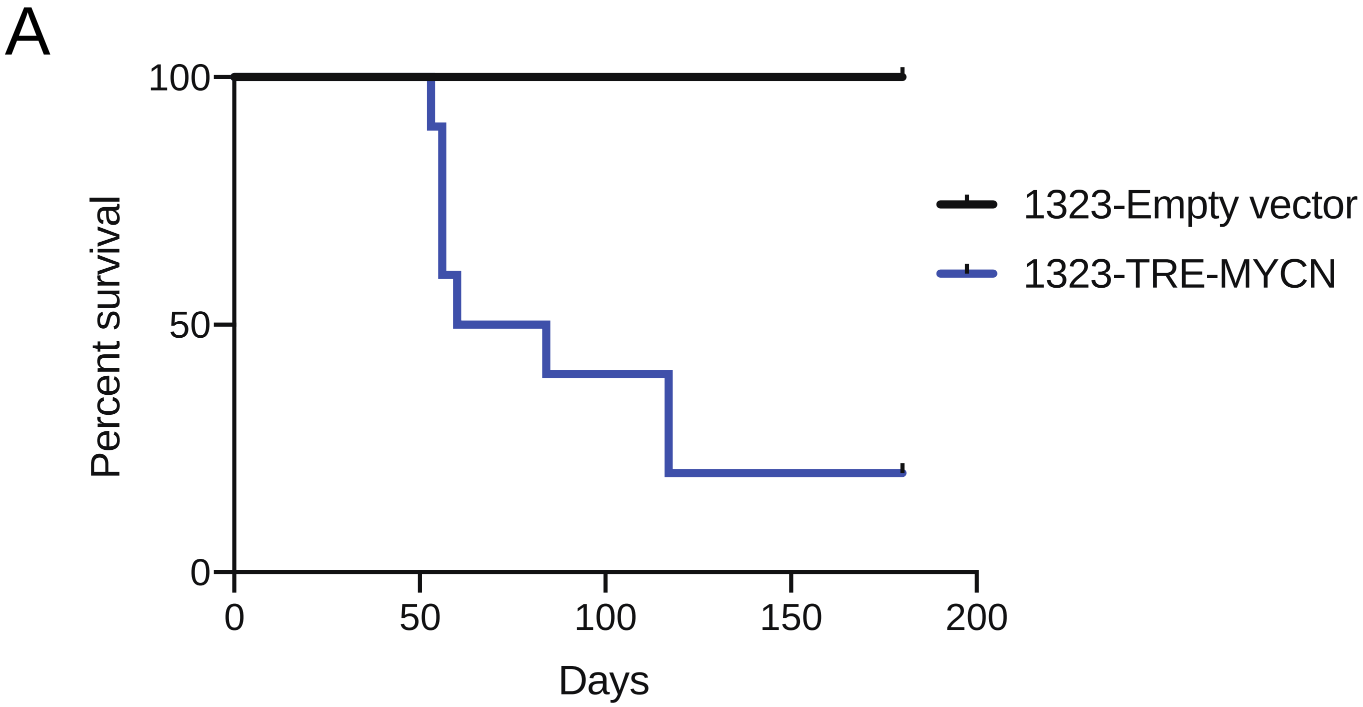
A
100
50
0
Percent survival
0
50
100
150
200
Days
1323-Empty vector
1323-TRE-MYCN

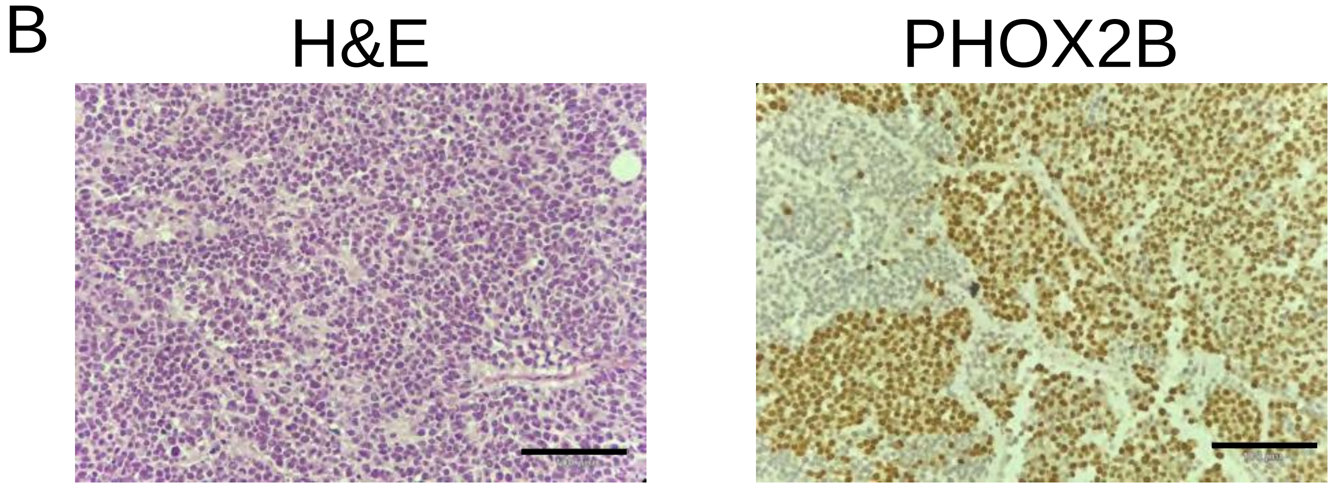
B
H&E
PHOX2B

Figure S3

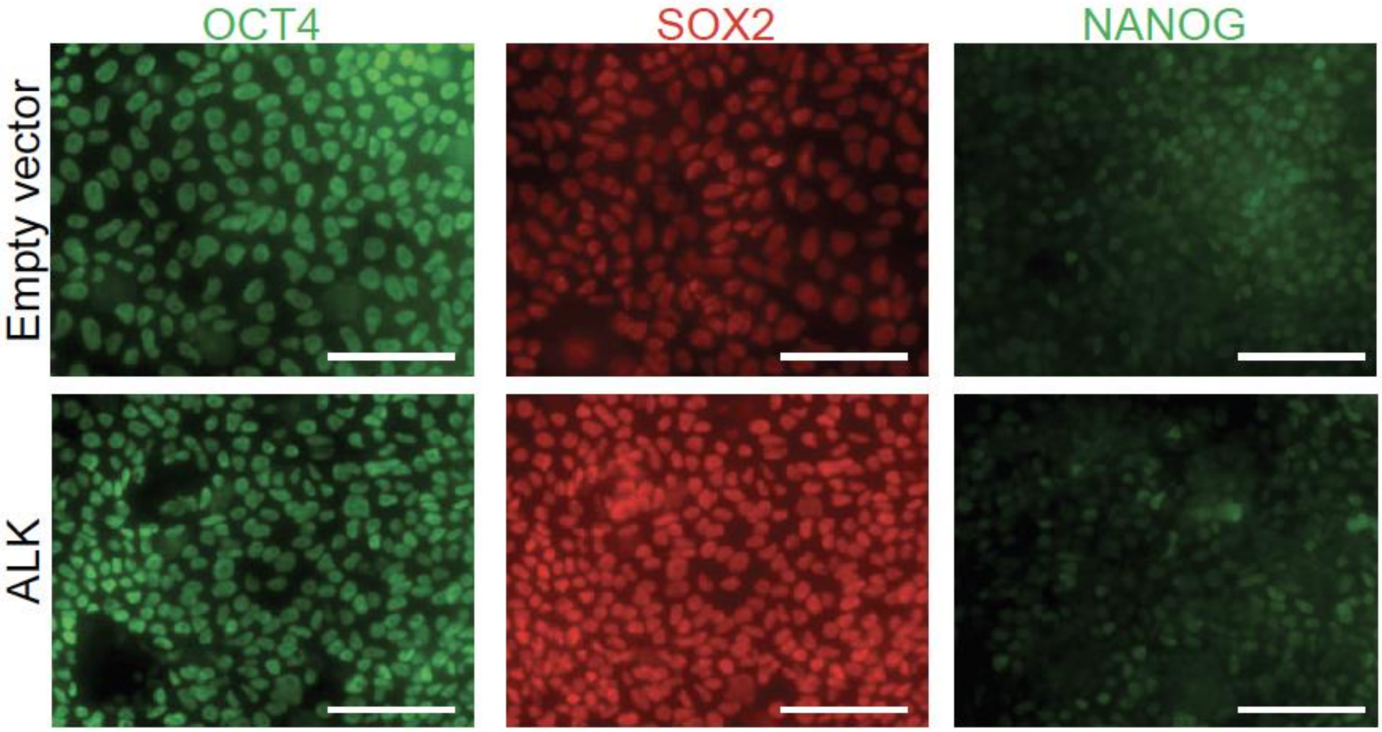

Figure S4

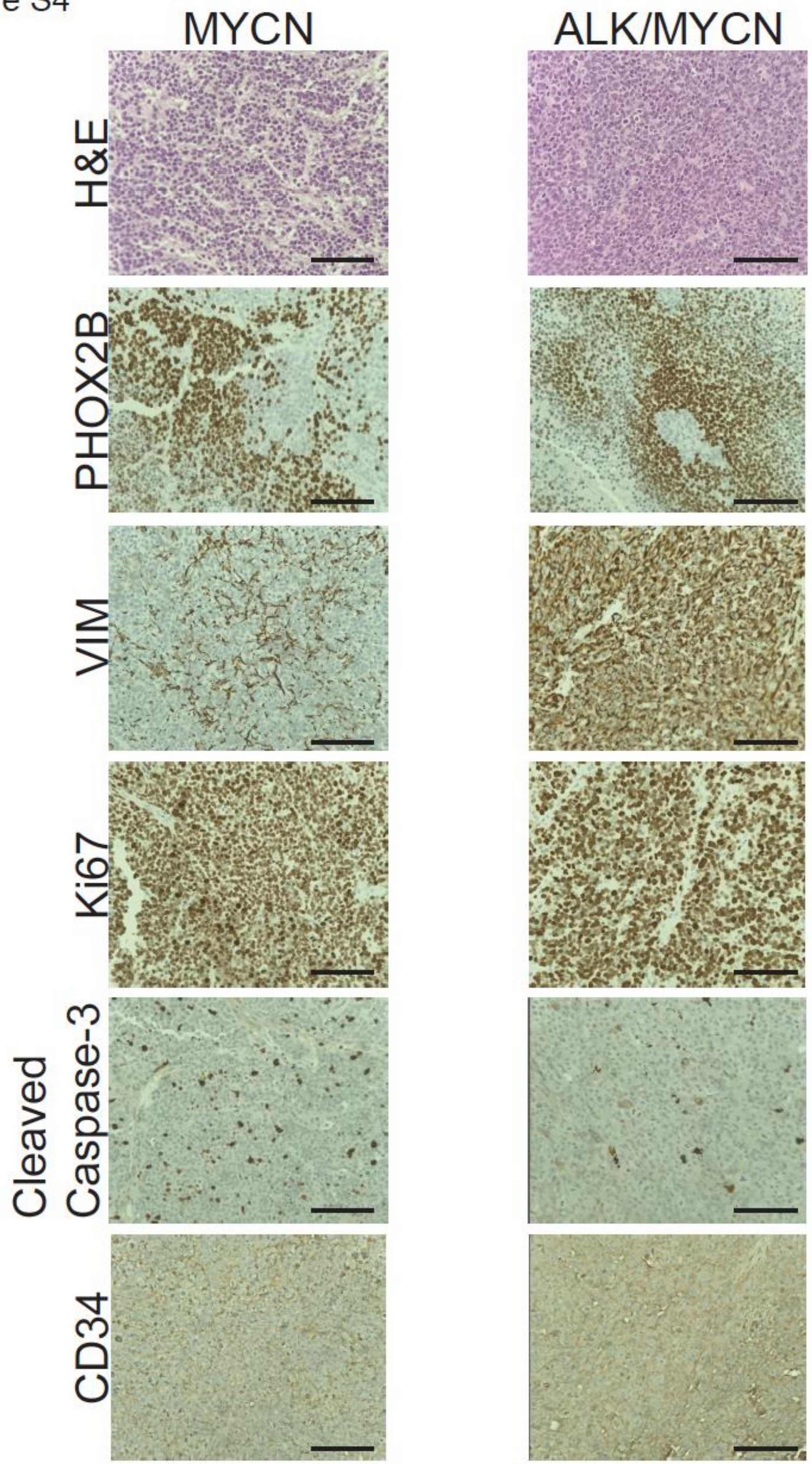

Figure S5

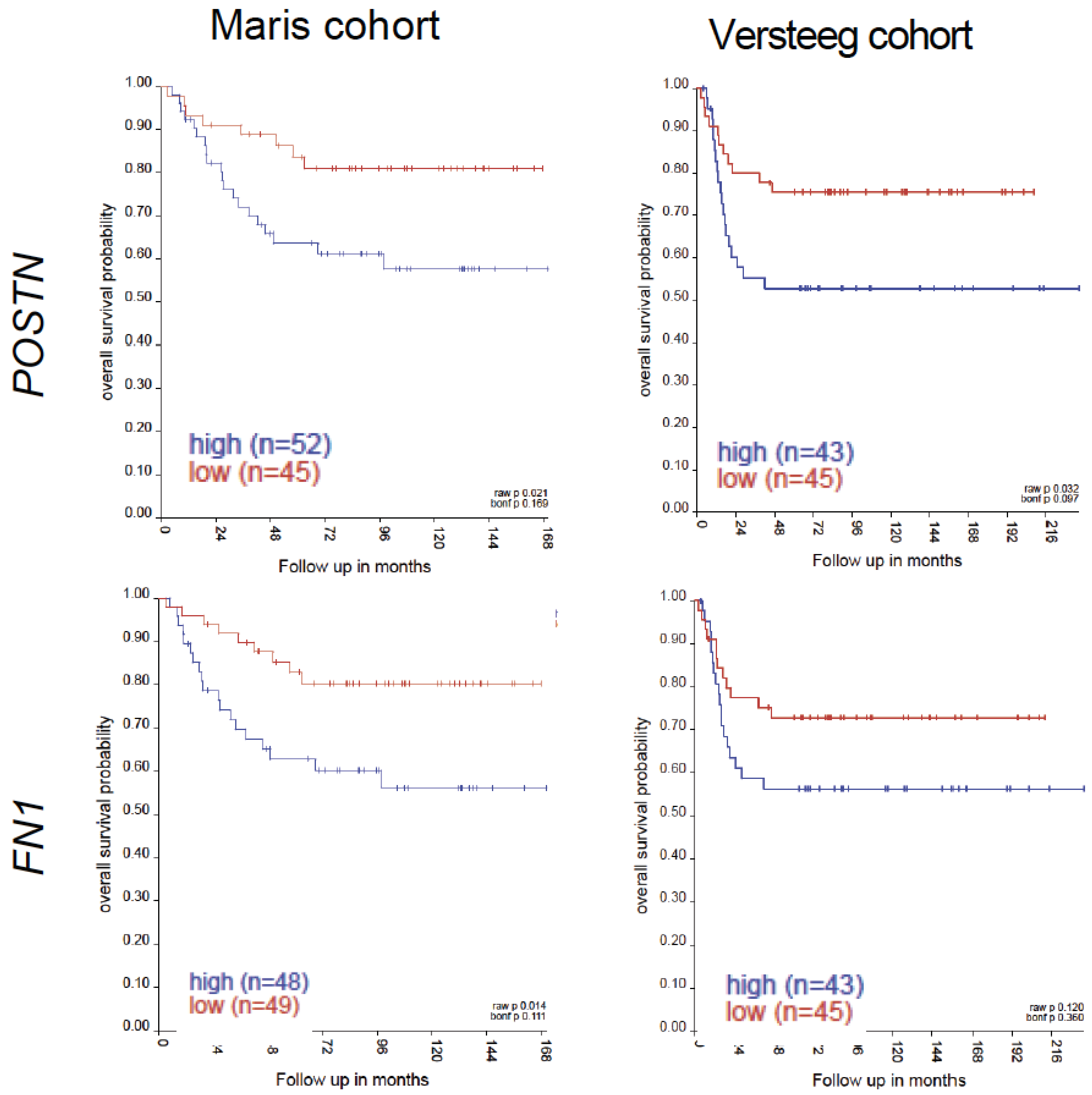

Maris cohort
Versteeg cohort
POSTN
FN1
overall survival probability
Follow up in months
high (n=52)
low (n=45)
raw p 0.021
bonf p 0.169
high (n=43)
low (n=45)
raw p 0.032
bonf p 0.097
high (n=48)
low (n=49)
raw p 0.014
bonf p 0.111
high (n=43)
low (n=45)
raw p 0.120
bonf p 0.360

Figure S6

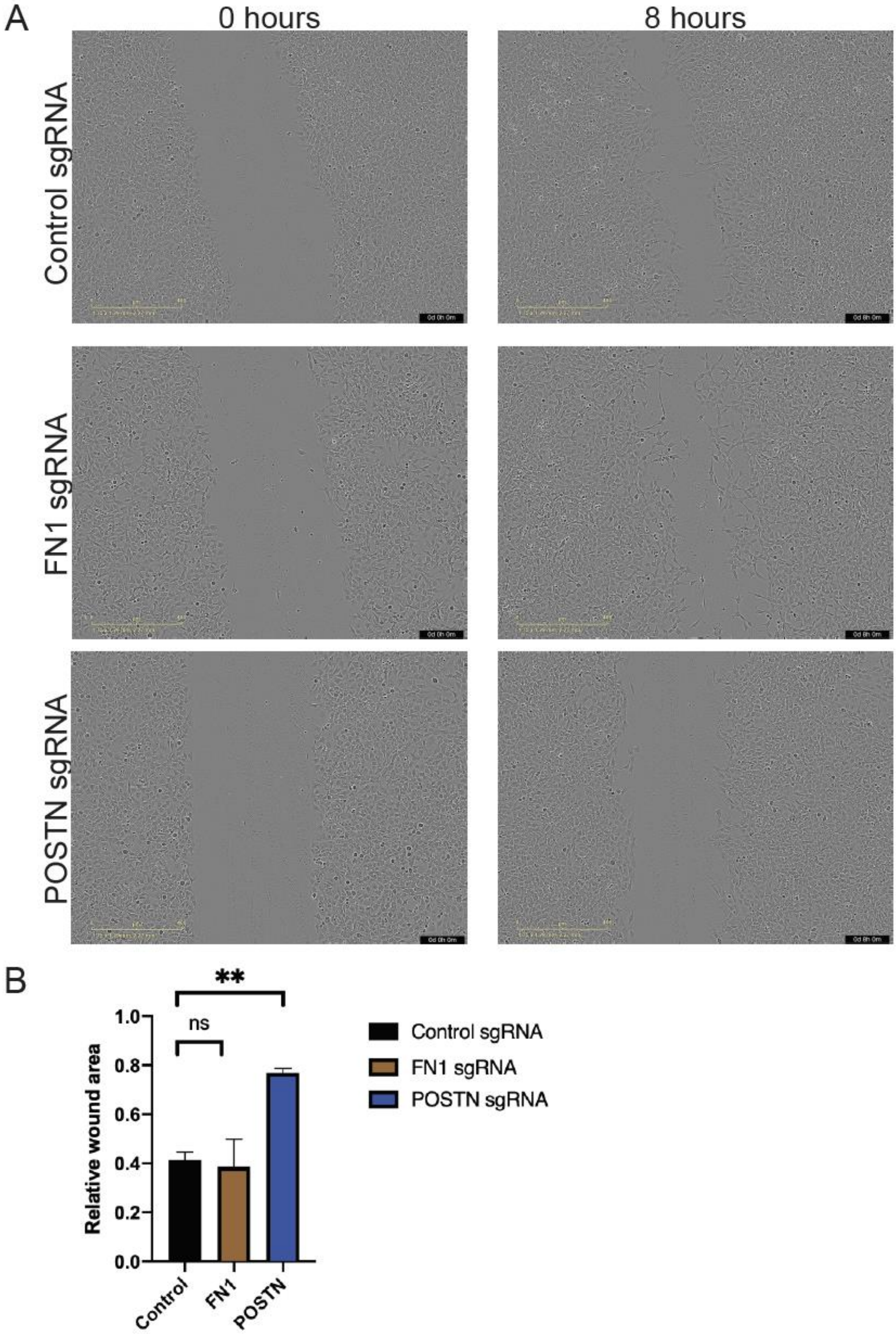

A
0 hours
8 hours
Control sgRNA
FN1 sgRNA
POSTN sgRNA
B
**
ns
Relative wound area
1.0
0.8
0.6
0.4
0.2
0.0
Control
FN1
POSTN
Control sgRNA
FN1 sgRNA
POSTN sgRNA

Figure S7

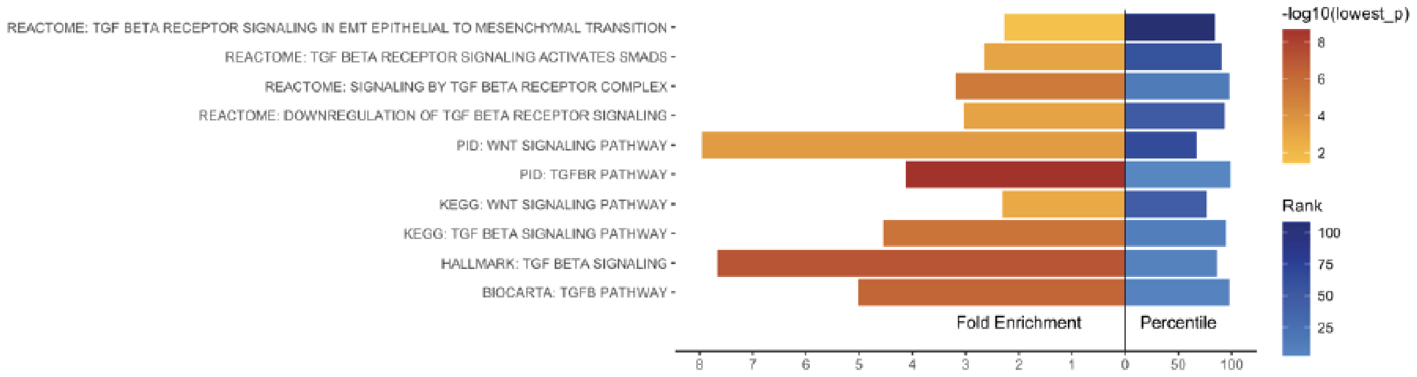

REACTOME: TGF BETA RECEPTOR SIGNALING IN EMT EPITHELIAL TO MESENCHYMAL TRANSITION
REACTOME: TGF BETA RECEPTOR SIGNALING ACTIVATES SMADS
REACTOME: SIGNALING BY TGF BETA RECEPTOR COMPLEX
REACTOME: DOWNREGULATION OF TGF BETA RECEPTOR SIGNALING
PID: WNT SIGNALING PATHWAY
PID: TGFBR PATHWAY
KEGG: WNT SIGNALING PATHWAY
KEGG: TGF BETA SIGNALING PATHWAY
HALLMARK: TGF BETA SIGNALING
BIOCARTA: TGFB PATHWAY
Fold Enrichment
Percentile
8 7 6 5 4 3 2 1 0 50 100
-log10(lowest_p)
8
6
4
2
Rank
100
75
50
25

Figure S8

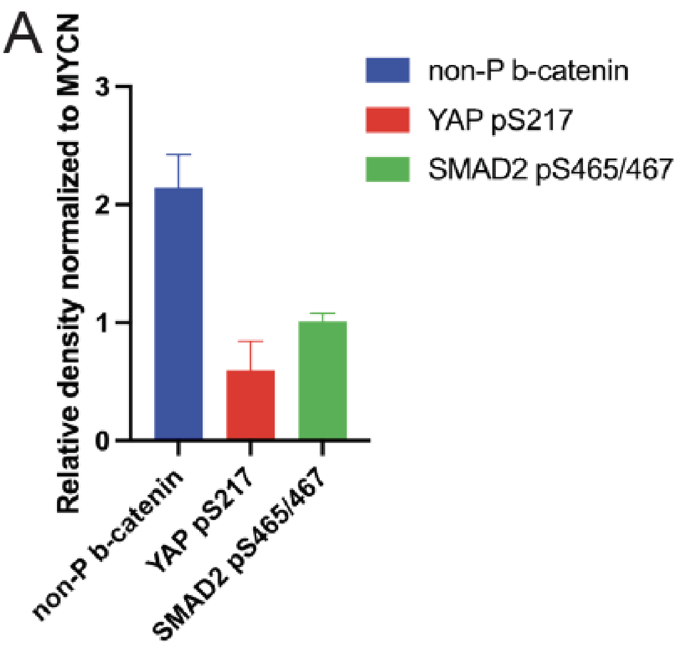
A
Relative density normalized to MYCN
3
2
1
0
non-P b-catenin
YAP pS217
SMAD2 pS465/467
non-P b-catenin
YAP pS217
SMAD2 pS465/467

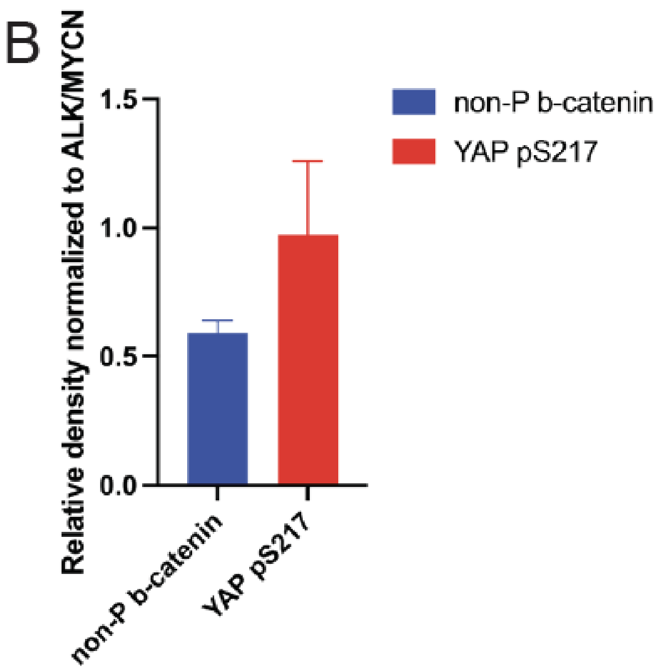
B
Relative density normalized to ALK/MYCN
1.5
1.0
0.5
0.0
non-P b-catenin
YAP pS217
non-P b-catenin
YAP pS217

Figure S9

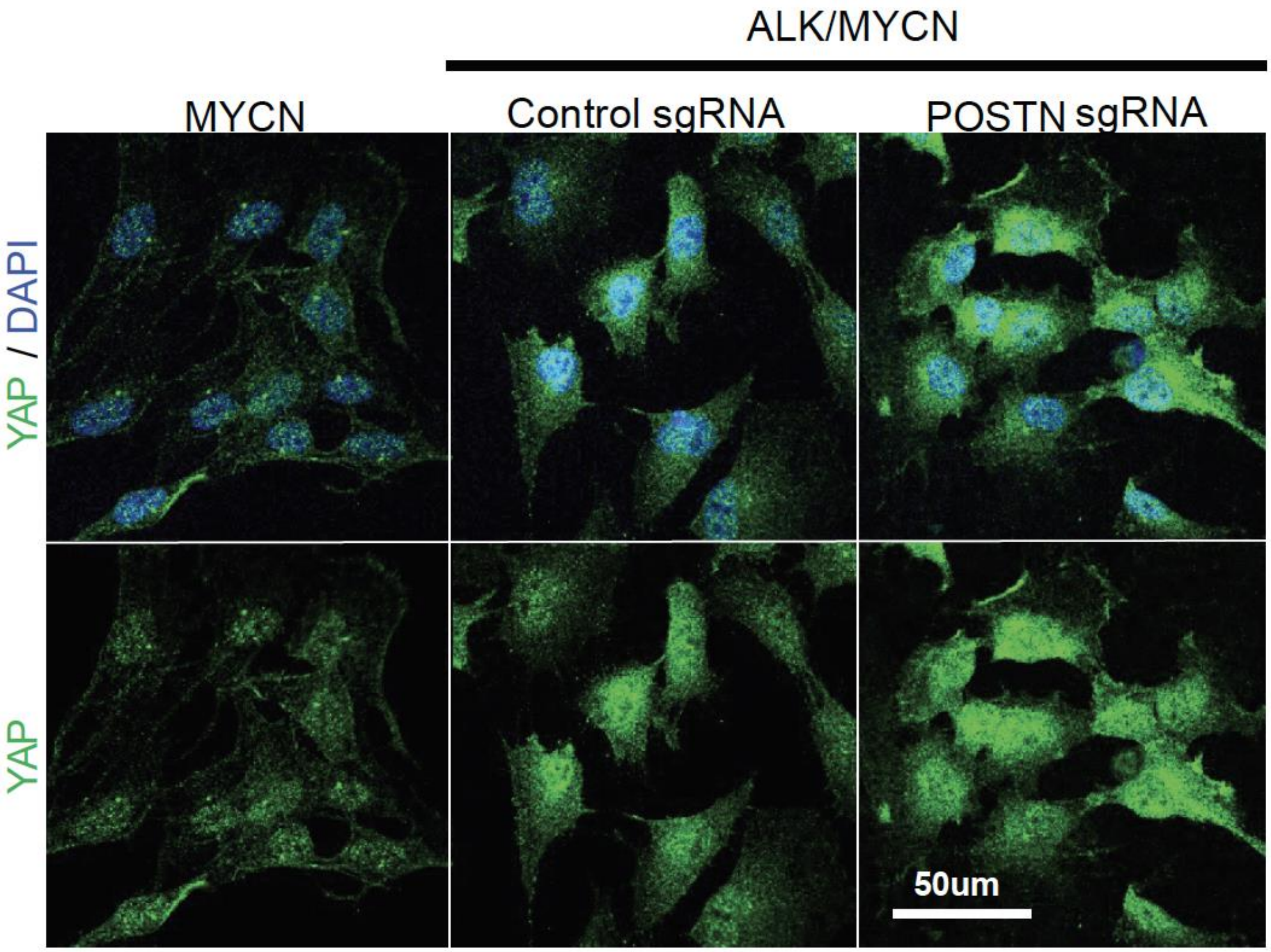

Figure S10

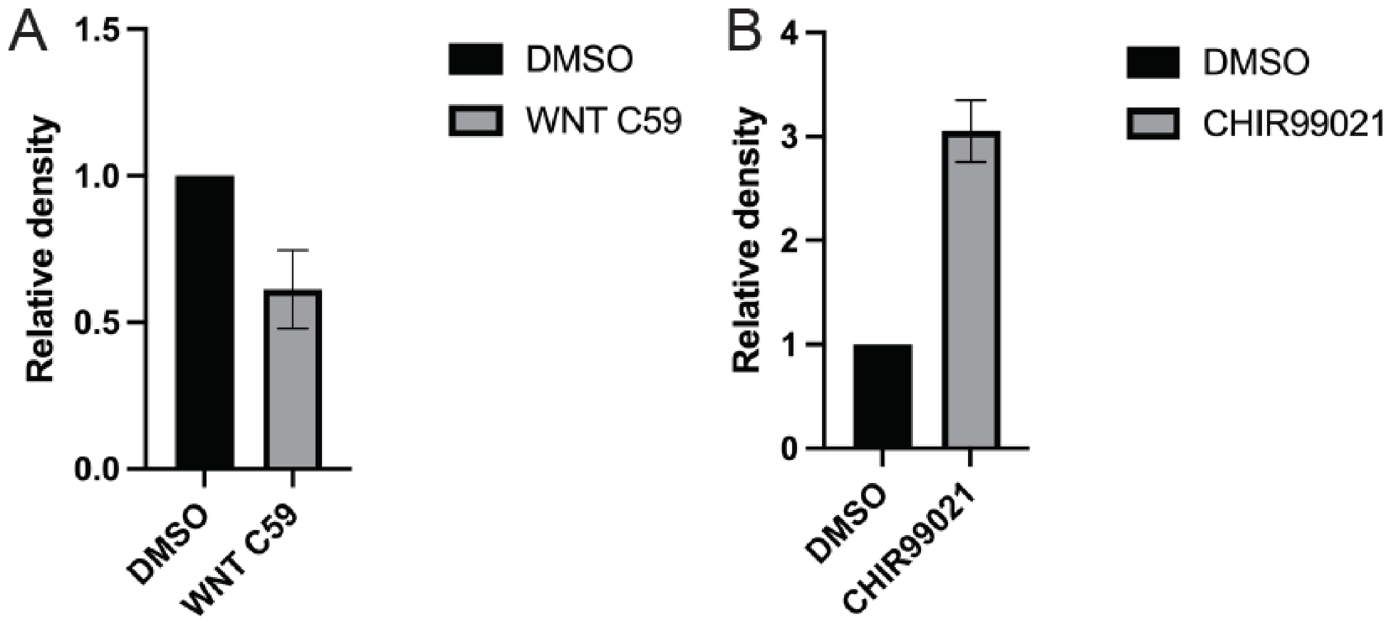
A
1.5
1.0
0.5
0.0
Relative density
DMSO
WNT C59
DMSO
WNT C59
B
4
3
2
1
0
Relative density
DMSO
CHIR99021
DMSO
CHIR99021
